# Developing a template matching algorithm for benchmarking hospital performance in a diverse, integrated healthcare system

**DOI:** 10.1097/MD.0000000000020385

**Published:** 2020-06-12

**Authors:** Daniel Molling, Brenda M. Vincent, Wyndy L. Wiitala, Gabriel J. Escobar, Timothy P. Hofer, Vincent X. Liu, Amy K. Rosen, Andrew M. Ryan, Sarah Seelye, Hallie C. Prescott

**Affiliations:** a VA Center for Clinical Management Research, Ann Arbor, MI; b Division of Research, Kaiser Permanente Northern California, Oakland, CA; c Department of Internal Medicine, University of Michigan; d VA Center for Healthcare Organization and Implementation Research, VA Boston Healthcare System, Boston, MA; e Department of Health Management and Policy, School of Public Health, University of Michigan, Ann Arbor, MI.

**Keywords:** health care research, mortality, outcomes research, quality of care

## Abstract

Template matching is a proposed approach for hospital benchmarking, which measures performance based on matching a subset of comparable patient hospitalizations from each hospital. We assessed the ability to create the required matched samples and thus the feasibility of template matching to benchmark hospital performance in a diverse healthcare system.

Nationwide Veterans Affairs (VA) hospitals, 2017.

Observational cohort study.

We used administrative and clinical data from 668,592 hospitalizations at 134 VA hospitals in 2017. A standardized template of 300 hospitalizations was selected, and then 300 hospitalizations were matched to the template from each hospital.

There was substantial case-mix variation across VA hospitals, which persisted after excluding small hospitals, hospitals with primarily psychiatric admissions, and hospitalizations for rare diagnoses. Median age ranged from 57 to 75 years across hospitals; percent surgical admissions ranged from 0.0% to 21.0%; percent of admissions through the emergency department, 0.1% to 98.7%; and percent Hispanic patients, 0.2% to 93.3%. Characteristics for which there was substantial variation across hospitals could not be balanced with any matching algorithm tested. Although most other variables could be balanced, we were unable to identify a matching algorithm that balanced more than ∼20 variables simultaneously.

We were unable to identify a template matching approach that could balance hospitals on all measured characteristics potentially important to benchmarking. Given the magnitude of case-mix variation across VA hospitals, a single template is likely not feasible for general hospital benchmarking.

## Abstract 2

Supplemental Digital Content is available in the text

## 1 Introduction

Template matching is a newer method of hospital benchmarking based on direct standardization. The method has been proposed by Silber et al^[1]^ as a potentially superior approach to the current practice of regression-based benchmarking. However, it has not been used operationally by any healthcare systems to our knowledge. The method uses multivariate matching to compare similar, matched samples of hospitalizations.^[1]^ First, a set of representative hospitalizations (the “template”) is identified from the healthcare system at large. Next, using multivariate matching, a sample of hospitalizations similar to the template hospitalizations is identified from each hospital. Finally, hospitals are compared directly based on the outcomes of these matched samples of hospitalizations.

Template matching may be considered as a fairer and more credible method for benchmarking than the current approach of indirect standardization using regression-based techniques, which compares how well hospital A does with its own patients relative to how an average hospital would be extrapolated to do with hospital A's patients (even if there was very little overlap between hospital A's patients and other hospital's patients).^[1]^ Because template matching compares all hospitals on the outcomes of a similar group of patients, it avoids extrapolations when there is limited overlap in hospital populations. Furthermore, hospitals can evaluate how closely their hospitalizations resemble the template hospitalizations, which may improve end-users’ understanding and trust of the benchmarking assessment.

While template matching has many attractive features, it is unclear whether it could be used for benchmarking overall hospital quality in a large, integrated healthcare system such as the Veterans Affairs (VA) healthcare system. Template matching requires that comparable hospitalizations can be identified across hospitals. For this reason, research studies using template matching have examined hospitalizations for common surgical procedures or medical diagnoses.^[1–4]^ Furthermore, to increase the pool of available hospitalizations for matching, studies have typically included hospitalizations from several years or excluded smaller-volume hospitals from the assessment.^[1–4]^ In practice, however, hospital systems typically prefer benchmarking assessments to use recent data, include all or many of the hospitalizations at each hospital, and evaluate smaller-volume hospitals.

We sought to assess the feasibility of template matching for yearly benchmarking of overall hospital performance in the nationwide VA healthcare system, where the diversity of patient case-mix and small case-volumes at many hospitals may pose particular challenges to implementing template matching. Specifically, we sought to evaluate the extent to which covariate balance could be achieved in matched samples of 300 hospitalizations from hospitals across the VA system.

## 2 Methods

### 2.1 Setting

The VA healthcare system is an integrated healthcare delivery system that provides comprehensive medical and psychiatric healthcare to veterans, and operates 134 acute care hospitals. VA hospitals had a median of 4544 hospitalizations in 2017 (range 267 to 16,253 across individual hospitals).^[5]^ VA hospitals vary in their critical care capabilities, as measured by availability of intensivist coverage and specialty services (e.g., surgery, cardiac catheterization laboratory).^[6,7]^ Critical care capability has been categorized on a scale of 1 (highest level of ICU care) to 4 (lowest level) or no ICU care.^[8]^

VA was among the first healthcare systems to have a universal electronic patient record, and to measure and report risk-adjusted mortality.^[9]^ The VA risk-adjustment model^[10–12]^ incorporates patient-level laboratory and physiological data collected within 24 hours of admission and performs as well as the Acute Physiology and Chronic Health Evaluation (APACHE IV)^[13]^ mortality model.

### 2.2 Overview of template matching approach

We followed the general template matching procedure proposed by Silber et al^[1]^ However, in contrast to prior studies by Silber and others that focused on a small number of common surgical procedures or medical diagnoses,^[1,4]^ and often considered multiple years of data,^[1,4,14]^ we examined hospital performance across a broader array of hospitalizations using data from a single calendar year to align with current VA hospital benchmarking practices.

Similar to Silber et al,^[1]^ we chose a template size of 300 to balance feasibility and power. While we did not limit our assessment to hospitalizations for specific diagnoses or surgical procedures, we did exclude hospitalizations for rare diagnoses and hospitals with fewer than 900 eligible hospitalizations in 2017, as described further below. We explored multiple matching algorithms and tested approaches using a single template as well as approaches using five templates (one for each of 5 tiers of hospitals, as defined by critical care capabilities).

### 2.3 Data source

We used the VA Inpatient Evaluation Center (IPEC) dataset from calendar year 2017, which contains clinical and administrative data for all VA hospitalizations, including laboratory and physiologic variables necessary for risk-adjustment. Specifically, available data included age, sex, race, ethnicity, 29 Elixhauser comorbidities,^[15]^ principal hospitalization diagnoses, an indicator for admission from the emergency department, an indicator for admission from a nursing facility, an indicator for major surgery within 24 hours of admission (surgical indicator), and 11 laboratory values drawn within the first 24 hours of hospitalization: sodium, blood urea nitrogen, glomerular filtration rate, glucose, albumin, bilirubin, white blood cell count, hematocrit, pH, PaCO_2_, and PaO_2_. We classified principal diagnoses into seven broad categories: cardiovascular, psychiatric and substance abuse, infection, gastrointestinal, respiratory, genitourinary/renal, and other (Supplemental Tables 1A–G;). As a measure of illness severity, we calculated predicted risk of 30-day mortality for each hospitalization using a logistic regression model,^[12]^ as in prior studies,^[16]^ that incorporates age, race/ethnicity, sex, surgical indicator, admission diagnoses (20 indicators for the 20 most common principal diagnosis categories using the Healthcare Cost and Utilization Project's single-level classification,^[17]^ which is sponsored by the agency for healthcare research and quality (ARRQ)), 29 Elixhauser comorbidities,^[15]^ and the 11 laboratory values drawn within the first 24 hours of hospitalization. The c-statistic for the predicted mortality model was 0.834.

### 2.4 Cohort exclusions

We excluded hospitalizations in which a patient was admitted as a transfer from another VA facility so that each hospitalization was considered only once. The original admitting hospital was considered the hospital of record, consistent with typical practice for hospital benchmarking. We excluded hospitalizations for organ transplantation, hospitalizations with a missing principal diagnosis code, and hospitalizations with a rare principal diagnosis code. We defined rare diagnoses pragmatically, as hospitalizations with a principal diagnosis category (healthcare cost and utilization project's single-level classification^[17]^) that occurred in fewer than 1 in 300 VA hospitalizations in 2017. We next excluded hospitals with fewer than 900 remaining eligible hospitalizations to guarantee a 3:1 matching ratio to the template. Finally, we excluded four hospitals with >90% psychiatric or substance-abuse-related admissions, as these hospitals had substantially different case-mix than the remaining 117 hospitals.

### 2.5 Examination of case-mix variation across facilities

To understand the degree of case-mix variation across VA hospitals, we measured patient characteristics at each hospital and reviewed the range of median values (or proportions) across hospitals for the primary case-mix variables used. We tested for differences across hospitals using the Kruskal–Wallis test for continuous variables and Pearson chi-square test for categorical variables. Kruskal–Wallis is a rank-based nonparametric test that assesses whether samples originate from the same distribution.^[18]^ Thus, it assesses for differences in the distribution of patient characteristics, not merely differences in median values.

### 2.6 Selection of the template

We sampled 300 hospitalizations from the overall population of eligible hospitalizations in the VA system (at random, without replacement) 1000 times to generate 1000 potential templates. We next selected the template that most closely approximated the overall population of hospitalizations on a subset of relevant variables: predicted probability of 30-day mortality, observed mortality, sex, race/ethnicity, surgical indicator, indicators for the 7 broad hospitalization diagnosis categories, and comorbidity indicators for congestive heart failure, chronic pulmonary disease, paralysis, renal failure, liver disease, metastatic cancer, and depression. Specifically, following the approach of Silber et al,^[1]^ we selected the template with the smallest Mahalanobis distance between the template mean and the VA system at-large. The Mahalanobis distance is a multivariate distance measure that takes into account correlated variables.^[19]^

We used the same process for selecting templates in the multi-template approach, but selected a separate template of 300 hospitalizations for each of 5 tiers of hospitals with similar critical care capabilities. In the multi-template approach, we also fixed the proportion of hospitalizations with a major surgery within 24 hours of admission (surgical hospitalizations) in each tier's template to be the same as the proportion of surgical hospitalizations for the median hospital in that tier. After fixing the ratio of surgical to non-surgical hospitalizations, we excluded hospitals with <3:1 match ratio for surgical and/or non-surgical hospitalizations (eg, for a template with 50 surgical and 250 non-surgical cases, a hospital would need to have at least 150 surgical hospitalizations and at least 750 non-surgical hospitalizations to be included in the assessment).

### 2.7 Matching to the template

After selecting the most representative template, we matched 300 hospitalizations from each hospital 1:1 to the 300 template cases. Hospitalizations were matched to the template via an optimal matching algorithm using the rcbalance package in R.^[20,21]^ We tested multiple matching algorithms, differing the specific variables used for matching, as well as the use of near-exact^[22]^ and fine balance^[23]^ constraints. Near-exact constraints require an exact match on the variable when possible but allow for a mismatch if an exact match is not possible. Fine balance ensures the same distribution of a variable between the template cases and matched hospitalizations; if a mismatch occurs for a specific categorical variable then the mismatch must be balanced by a reciprocal mismatch in the opposite direction for another pair of matched hospitalizations.

We initially tested matching algorithms using all available variables; in subsequent runs, we limited to progressively smaller subsets of variables believed to have greater prognostic significance. We present 10 representative matching algorithms in this manuscript, which we refer to as “matching runs.” Statistical code for selected runs is presented at https://github.com/CCMRcodes/TemplateMatching02/.

### 2.8 Assessing quality of matches

We assessed match quality using 2 approaches. First, we performed a cross-match test to assess the overall quality of each hospital's match to the template, using the variables included in the matching algorithm.^[1,24]^ Conceptually, the cross-match test combines the hospitalizations from the template and a given hospital into a single dataset, and then divides the dataset into pairs such that the total Mahalanobis distance within pairs is minimized. A *P* value is generated based on the number of times a template case is paired with a non-template hospitalization. A low *P* value indicates that template cases are being paired to each other rather than to hospitalizations from the hospital under evaluation, and thus the hospital under evaluation is poorly matched to the template. For our purposes we did not exclude poorly matched hospitals from later analysis. Additionally, we assessed the variation in patient characteristics across matched samples using Kruskal–Wallis^[18]^ or chi-square tests.

### 2.9 Benchmarking hospitals

After matching hospitalizations from each hospital to the template, we ranked hospitals based on 30-day mortality rates for the matched hospitalizations, which were estimated using hierarchical logistic regression. We took advantage of the matched data by clustering on the template matched patient and adjusted for predicted probability of 30-day mortality. We then used each hospital's estimated fixed effect to rank their relative performance.

### 2.10 Similarity of hospital rankings across template matching runs

To understand whether different matching runs yielded meaningfully different benchmarking assessments, we categorized hospitals as top (top quintile), median (middle 3 quintiles), or bottom (bottom quintile) performers. We then tested for differences in performance category assignment using a chi-square test, similar to Hu et al.^[4]^

### 2.11 Similarity of hospital ranking by template matching vs regression

To understand how template matching performance rankings compared to conventional regression, we estimated a hierarchical logistic regression model using the entire dataset after adjusting for predicted risk of 30-day mortality, as in prior work.^[25]^ We then tested for differences in performance category assignment by regression versus template matching using a chi-square test.

The study was approved by the Ann Arbor VA Institutional Review Board.

## 3 Results

**Figure 1:**
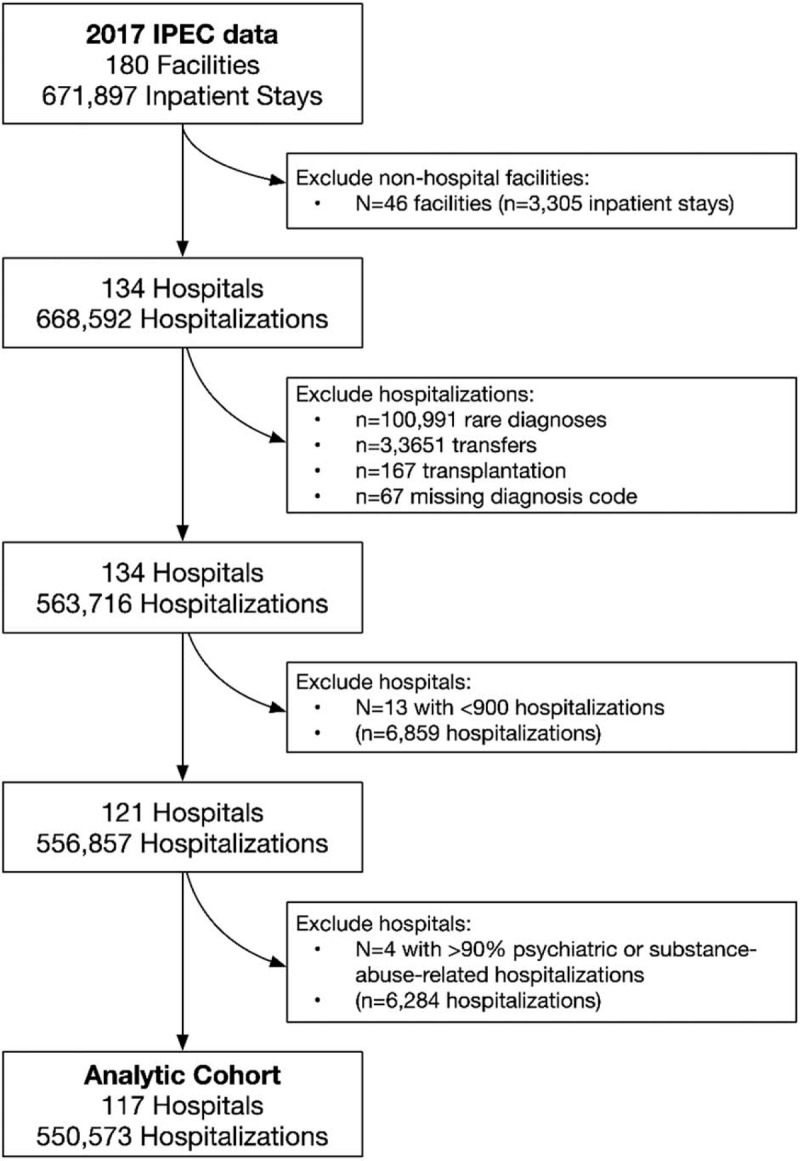
Consort Diagram showing the inclusion of hospitals and hospitalizations in the study.

Among 668,592 hospitalizations at 134 VA facilities in 2017, 550,573 (82%) hospitalizations at 117 (87%) VA hospitals met inclusion criteria (Fig. 1). We excluded 100,991 (15.1%) hospitalizations with a rare principal diagnosis, 167 (0.02%) hospitalizations for organ transplantation, and 67 (0.01%) hospitalizations with a missing diagnosis code. We excluded 3651 transfers from VA facilities (0.5%). Finally, we excluded 6284 (0.9%) hospitalizations at 13 hospitals with fewer than 900 hospitalizations and 4 hospitals with ≥90% psychiatric or substance-abuse-related hospitalizations. Our final cohort included 72 principal diagnosis categories. The 10 most common were alcohol-related disorders, mood disorders, congestive heart failure, nonspecific chest pain, cardiac dysrhythmias, coronary atherosclerosis, sepsis, COPD, hypertension with complications, and substance-related disorders. The full list of principal diagnosis categories is presented in Supplemental Tables 1A–G.

### 3.1 Case-mix variation across hospitals

After applying the above exclusions, substantial case-mix variation persisted across the 117 VA hospitals. The distributions of all patient characteristics differed in clinically substantive ways across hospitals, for example with rates of comorbidities increasing by 50% to 100% across the interquartile range (Table 1). Other notable differences included median age that ranged from a minimum of 57 to a maximum of 75 years across hospitals; percent surgical hospitalizations that ranged from 0.0% to 21.0%; percent of admissions through the emergency department that ranged from 0.1% to 98.7%; and percent Hispanic patients that ranged from 0.2% to 93.3% (Table 1).

**Table 1:**
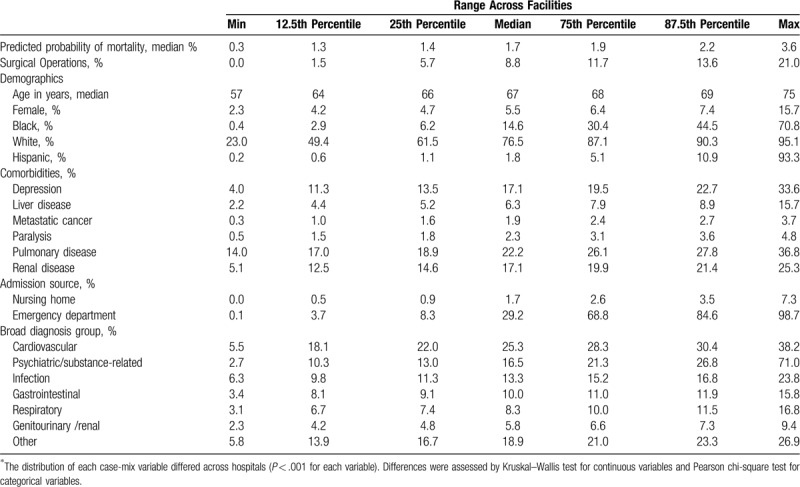
Case-mix variation across unmatched hospitalization populations at 117 VA hospitals.

While the variation in principal diagnosis category was reduced after the exclusion of hospitals with predominantly psychiatric admissions, differences in principal diagnosis category across hospitals nonetheless remained significant (Table 1, Supplemental Table 2;). For example, the percentage of cardiovascular admissions ranged from a minimum of 5.5% to a maximum of 38.2%, while the percentage of psychiatric or substance-abuse-related hospitalizations ranged from 2.7% to 71.0%.

Hospitals within the 5 tiers of critical care capabilities were more homogenous, but the differences across hospitals remained clinically and statistically significant (Supplemental Tables 3A–E;). For example, among tier 1 hospitals, percent surgical hospitalizations ranged from 0.0% to 20.2% (*P* < .001); percent of admissions through the emergency department ranged from 0.2% to 98.3% (*P* < .001); and percent Hispanic patients ranged from 0.4% to 93.3% (*P* < .001) (Supplemental Table 3A;).

### 3.2 Evaluation of template matching approaches – using a single template

Ten representative template matching runs are presented in Table 2 and Supplemental Table 4. The initial matching algorithms, which attempted to match hospitalizations to template cases using 56 to 121 matching variables, frequently failed to converge (Table 2, runs 1, 3). Subsequent algorithms using 35 to 56 variables converged (Table 2, runs 2, 5, 6), but resulted in poor balance on many of the matching variables (9–14 of 35 variables), and 16% to 18% of hospitals were poorly matched to the template (i.e., failed the cross-match test). Interestingly, small modifications to the matching algorithm—such as changing near-exact or fine balance constraints on a single variable (Table 2, match runs 4, 5)—changed whether a matching algorithm was able to converge.

**Table 2:**
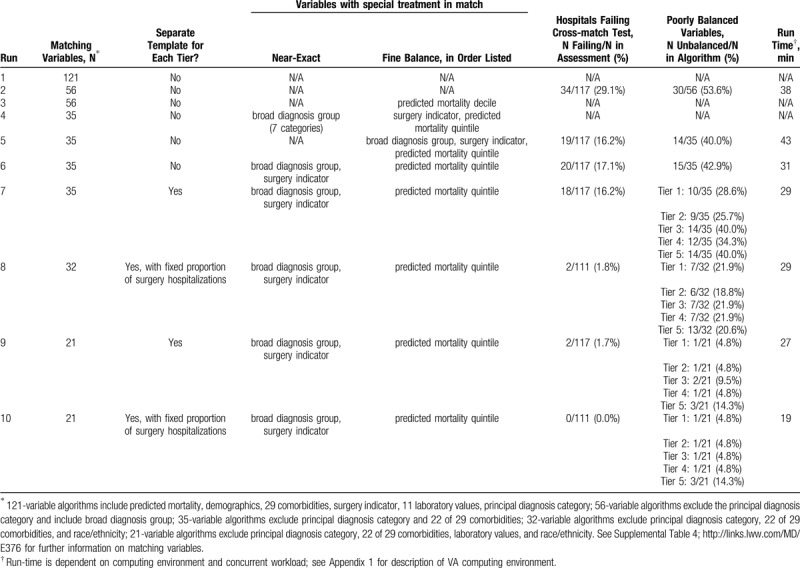
Methods and outcomes for 10 representative template matching runs.

### 3.3 Evaluation of template matching approaches – using several templates

Using a separate template for each of 5 tiers of hospitals (Table 2, match run 7) resulted in a similar number of poorly matched hospitals and variables. After limiting the matching algorithm to a small subset of variables deemed to have the highest prognostic significance based on clinical experience: predicted mortality, age, sex, admission source, 7 major comorbidities, surgical indicator, broad hospitalization diagnosis category (Table 2, match run 9), more than 98% of hospitals were well-matched to the template and fewer variables (6–7 for tiers 1–4) were poorly balanced.

In matching runs 8 and 10, we fixed the proportion of surgical hospitalizations in the template to the median proportion of surgical hospitalizations among hospitals in each tier (Table 2). In these runs, an additional 6 hospitals with insufficient surgical hospitalizations to guarantee a 3:1 match ratio were excluded. Fixing the proportion of surgical hospitalizations resulted in fewer unbalanced variables to (e.g., 6–7 unmatched variables per tier for run 8). When we also excluded laboratory values from our matching algorithm (run 10), all hospitals were well matched to the template and only a single matching variable (percent admission from emergency department) was poorly balanced across hospital tiers 1–4 (Table 3, Supplemental Table 5;).

**Table 3:**
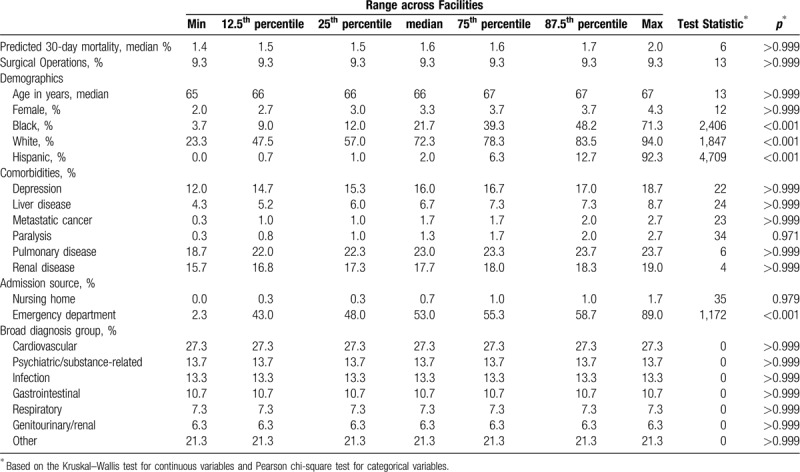
Case-mix variation across matched hospitalization cohorts for 55 Tier 1 hospitals, using matching run 10.

**Figure 2:**
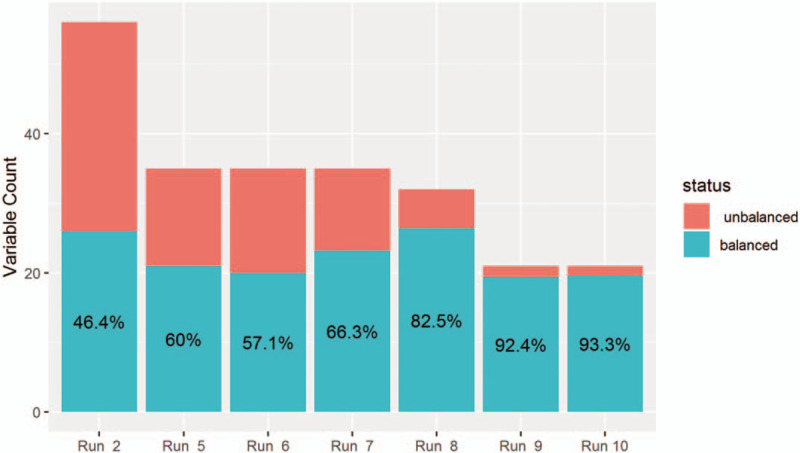
Number of matching variables and percent balanced post-match for completed template matching runs. Legend: This stacked bar graph depicts the number of matching variables (y-axis) that were balanced (turquoise) and unbalanced (orange) for each completed template matching run (x-axis). For runs 7–10, in which separate templates were used for each of 5 tiers of hospitals, the average number of matched variables across the 5 tiers is displayed.

In general, using a tiered approach and a smaller set of matching variables resulted in a higher percentage of well-balanced variables after matching (Fig. 2). When using 35 variables, 57.1% of the variables were well-balanced after matching when using the entire dataset (Fig. 2, run 6). Using the same variables but a tiered approach resulted in an average of 66.3% of variables being matched across tiers (Fig. 2, run 7). When using a tiered approach with a fixed proportion of surgical hospitalizations and 21 variables, 93.3% of the variables were well-balanced after matching (Fig. 2, run 10).

### 3.4 Similarity of hospital rankings by different benchmarking assessments

There were substantial differences in benchmarking assessments generating from the different template matching runs (Supplemental Fig. 1;), and from template matching vs regression (Supplemental Figs. 2 and 3;). For example, when comparing regression to template matching run 10, there were significant differences in hospital performance assessments for 4 of 5 hospital tiers (Supplemental Fig. 2;). When laboratory values were included in the template matching algorithm (Table 2, run 8, Fig. 2, run 8), match quality was lower (i.e., greater imbalance among matching variables, two hospitals failed the cross-match test), and the difference in performance assessments was more pronounced (Supplemental Fig. 3;).

## 4 Discussion

In this study, we evaluated a number of matching algorithms and approaches to implementing template matching for benchmarking 30-day mortality across VA hospitals. In contrast to prior studies, and to align with current VA benchmarking practices, we tested template matching using hospitalizations from a single calendar year and did not limit our approach to specific hospitalization diagnoses.

We were unable to achieve the overarching goal of template matching, which is to identify matched samples of hospitalizations that have similar characteristics, such that differences in outcomes across the matched samples can be assumed to be due to differences in hospital quality. Rather, we found that the magnitude of case-mix variation across VA hospitals precludes the use of a single template, or even a small collection of templates.

While we could balance matched hospital samples on some important characteristics (e.g., major comorbidities, predicted 30-day mortality), other variables often considered of prognostic significance could not be balanced in any algorithm (e.g., percent admission via emergency department), or could not be balanced simultaneously with other important characteristics (e.g., admission laboratory values). In general, the matching algorithms could balance around 20 variables. The more variables included, the less control we had over which 20 variables were balanced. The partial balance achieved in matching hospital samples was not sufficient for benchmarking purposes, as performance assessments differed across the various matching algorithms tested.

While a more homogeneous hospital system may be better able balance a larger number of variables, we do not believe these challenges are unique to the VA system. Other multi-hospital systems with case-mix variation across hospitals are likely to face similar challenges in implementing template matching for benchmarking overall hospital performance.

It is important to note that the lack of comparable patient populations does not suggest that regression-based benchmarking is better or fairer than template matching. Rather, our findings reinforce the problem of regression-based performance assessment, as we show definitively that the benchmarks against which any hospital is measured are based on substantially extrapolated estimates of how the average hospital would perform.

While we were unable to implement template matching for benchmarking overall hospital performance in the VA system, we still believe that matching can be used to enhance the fairness of hospital performance assessment. One option we considered was to match hospitalizations on a smaller number of prognostic scores rather than individual variables (e.g., matching on a “comorbidity score” rather than individual comorbidities; an “acute physiology score” rather than individual laboratory values). This type of approach would improve the feasibility of matching, but is less transparent than matching on individual characteristics and requires end-users to trust the multivariable scores. Furthermore, it is possible to paradoxically worsen the balance of unmeasured variables when matching on multivariable scores.^[26]^ For these reasons, we have not pursued template matching by prognostic scores, but this approach could be explored in future work.

Second, we are now pursuing hospital-specific template matching.^[2]^ Under this approach, also proposed by Silber et al, a sample of hospitalizations is selected from each hospital under evaluation; a set of similar hospitalizations is then identified from each comparison hospital. A main benefit of this approach is that the template is personalized to each hospital, so each hospital's performance assessment is based on outcomes for hospitalizations that well-approximate their typical case-mix. A second benefit of this approach is that the comparison (the hospitals against which each hospital are compared) are also customized, ensuring that hospitals are compared only to those hospitals who care for similar patients. Because not every hospital is included in the comparison, it is possible to ensure that performance assessments are based only on high-quality matches. Additionally, this approach overcomes the need to exclude hospitals without surgical services, hospitals with small case-volumes, and hospitals with predominantly psychiatric or substance-abuse-related hospitalizations. Thus, we suspect that a hospital-specific template^[2]^ may be more feasible for operational use in the VA.

Hospital-specific template matching provides different information than the single template approach or regression-based performance assessment. Because each hospital's assessment is customized (different template, and different comparisons), it cannot yield hospital rankings—but instead answers the question “how well does hospital A do for the patients it typically sees compared to other hospitals who care for similar patients?”.^[2]^ While the lack of hospital rankings may be viewed as a limitation by some, our study has led us to question the validity of such rankings. It is not clear that any method can address the problem of case-mix variation to yield fair or meaningful hospital rankings.

There are some limitations to our study. First, some of the measured case-mix variation in our study may reflect differences in hospital structure and organization rather than patient physiology. For example, we suspect that the wide variation in percent admissions through the ER is explained—in part—by differences in ER capacity and hospital admission practices across hospitals (e.g., the relative use of direct admissions vs referral to the ER for admission). Second, while we explored multiple matching algorithms, our approaches were not exhaustive. We focused on matching individual pairs of hospitalizations directly. It is possible that alternative matching approaches, using composite scores or machine learning algorithms may be better able to balance the samples of hospitalizations. However, we remain concerned that such approaches could exacerbate imbalance of unmeasured characteristics, are less transparent than direct matching, and cannot overcome extremely imbalanced variables (e.g., percentage of admissions via emergency department) so have instead decided to pursue hospital-specific template matching in our future work. Third, it is possible that a single template approach may be feasible if using multiple years of data. However, we did not examine this as it would be less suitable for operational use in the VA system.

## 5 Conclusions

There is substantial case-mix variation across VA hospitals, most notably in patient age, percent admissions through the emergency department, percent psychiatric or substance-abuse-related hospitalizations, and race/ethnicity. Case-mix variation persisted even after applying several exclusions. We were unable to identify a template matching algorithm that balanced hospitals on all measured characteristics potentially important to benchmarking. Given the magnitude of case-mix variation across VA hospitals, a single template was not feasible for general hospital benchmarking.

## Author contributions

Analysis: Molling.

Conceptualization: All authors.

Funding Acquisition: Prescott.

Writing (original draft): Molling, Seelye, Prescott

Writing (review & editing for critical intellectual content): All authors.

## Supplementary Material

Supplemental Digital Content

## References

[1] Silber JH Rosenbaum PR Ross RN Template matching for auditing hospital cost and quality. Health Serv Res 2014;49:1446–74. 24588413 10.1111/1475-6773.12156 PMC4213044

[2] Silber JH Rosenbaum PR Ross RN A hospital-specific template for benchmarking its cost and quality. Health Serv Res 2014;49:1475–97. 25201167 10.1111/1475-6773.12226 PMC4213045

[3] Silber JH Rosenbaum PR Wang W Auditing practice style variation in pediatric inpatient asthma care. JAMA Pediatr 2016;170:878–86. 27398908 10.1001/jamapediatrics.2016.0911

[4] Hu W Chan CW Zubizarreta JR Incorporating longitudinal comorbidity and acute physiology data in template matching for assessing hospital quality: an exploratory study in an integrated health care delivery system. Medical Care 2018;56:448–54. 29485529 10.1097/MLR.0000000000000891

[5] Wang XQ Vincent BM Wiitala WL Veterans Affairs patient database (VAPD 2014-2017): building nationwide granular data for clinical discovery. BMC Med Res Methodol 2019;19:94. 31068135 10.1186/s12874-019-0740-x PMC6505066

[6] Haupt MT Bekes CE Brilli RJ Guidelines on critical care services and personnel: recommendations based on a system of categorization of three levels of care. Crit Care Med 2003;31:2677–83. 14605541 10.1097/01.CCM.0000094227.89800.93

[7] Brilli RJ Spevetz A Branson RD Critical care delivery in the intensive care unit: defining clinical roles and the best practice model. Crit Care Med 2001;29:2007–19. 11588472 10.1097/00003246-200110000-00026

[8] Almenoff P Sales A Rounds S Intensive care services in the Veterans Health Administration. Chest 2007;132:1455–62. 17925432 10.1378/chest.06-3083

[9] Fihn SD Francis J Clancy C Insights from advanced analytics at the Veterans Health Administration. Health Aff (Millwood) 2014;33:1203–11. 25006147 10.1377/hlthaff.2014.0054

[10] Render ML Welsh DE Kollef M Automated computerized intensive care unit severity of illness measure in the Department of Veterans Affairs: preliminary results. SISVistA Investigators. Scrutiny of ICU Severity Veterans Health Sysyems Technology Architecture. Crit Care Med 2000;28:3540–6. 11057814 10.1097/00003246-200010000-00033

[11] Render ML Kim HM Welsh DE Automated intensive care unit risk adjustment: results from a National Veterans Affairs study. Crit Care Med 2003;31:1638–46. 12794398 10.1097/01.CCM.0000055372.08235.09

[12] Render ML Deddens J Freyberg R Veterans Affairs intensive care unit risk adjustment model: validation, updating, recalibration. Crit Care Med 2008;36:1031–42. 18379226 10.1097/CCM.0b013e318169f290

[13] Cerner. APACHE Outcomes; 2018. Available at: https://apacheout comes.cernerworks.com/criticaloutcomeshome/. Accessed December 17, 2018.

[14] Ridgeway G Norgaard M Rasmussen TB Benchmarking Danish hospitals on mortality and readmission rates after cardiovascular admission. Clin Epidemiol 2019;11:67–80. 30655706 10.2147/CLEP.S189263 PMC6324920

[15] van Walraven C Austin PC Jennings A A modification of the Elixhauser comorbidity measures into a point system for hospital death using administrative data. Medical Care 2009;47:626–33. 19433995 10.1097/MLR.0b013e31819432e5

[16] Prescott HC Kepreos KM Wiitala WL Temporal changes in the influence of hospitals and regional healthcare networks on severe sepsis mortality. Crit Care Med 2015;43:1368–74. 25803652 10.1097/CCM.0000000000000970 PMC4470811

[17] HCUP. Beta Clinical Classifications Software (CCS) for ICD-10-CM/PCS. https://www.hcup-us.ahrq.gov/toolssoftware/ccs10/ccs10.jsp. [access date September 11, 2019].

[18] Kruskal W Wallis W Use of ranks in one-criterion variance analysis. J Am Stat Assoc 1952;47:583–621.

[19] Mahalanobis PC On the generalized distance in statistics. Proc Natl Inst Sci India 1936;2:49–55.

[20] Pimentel SD Kelz RR Silber JH Large, sparse optimal matching with refined covariate balance in an observational study of the health outcomes produced by new surgeons. J Am Stat Assoc 2015;110:515–27. 26273117 10.1080/01621459.2014.997879 PMC4531000

[21] Pimentel SD. rcbalance: Large, Sparse Optimal Matching with Refined Covariate Balance. https://cran.r-project.org/web/packages/rcbalance/index.html. [access date September 11, 2019].

[22] Zubizarreta JR Reinke CE Kelz RR Matching for several sparse nominal variables in a case-control study of readmission following surgery. Am Stat 2011;65:229–38. 25418991 10.1198/tas.2011.11072 PMC4237023

[23] Rosenbaum P Ross R Silber J Minimum distance matched sampling with fine balance in an observational study of treatment of ovarian cancer. J Am Stat Assoc 2007;102:75–82.

[24] Heller R, Small D, Rosenbaum p. crossmatch. The Cross-match Test. https://cran.r-project.org/web/packages/crossmatch/index.html. Published 2012. [access date September 11, 2019].

[25] Vincent BM Wiitala WL Luginbill KA Template matching for benchmarking hospital performance in the Veterans Affairs healthcare system. Medicine (Baltimore) 2019;98:e15644. 31096485 10.1097/MD.0000000000015644 PMC6531221

[26] Brooks JM Ohsfeldt RL Squeezing the balloon: propensity scores and unmeasured covariate balance. Health Serv Res 2013;48:1487–507. 23216471 10.1111/1475-6773.12020 PMC3725536

