# Supplementary material for: Developing a template matching algorithm for benchmarking hospital performance in a diverse, integrated healthcare system
Source: Medicine (Baltimore). 2020 Jun 12;99(24):e20385. doi: 10.1097/MD.0000000000020385 (PMC7302661; doi:10.1097/MD.0000000000020385)
Supplement: Supplemental Digital Content [file medi-99-e20385-s001.docx]

**Online Supplement to** Developing a Template Matching Algorithm for Benchmarking Hospital Performance in the Nationwide Veterans Affairs System

**Authors**: Daniel Molling, MS; Brenda Vincent, MS; Wyndy L. Wiitala, PhD; Gabriel J. Escobar, MD; Timothy Hofer, MD, MSc; Theodore J. Iwashyna, MD, PhD; Vincent X Liu, MD, MS; Kaitlyn Luginbill, MPH; Amy K. Rosen, PhD; Michael Shwartz, PhD, MBA; Andrew M. Ryan, PhD; Sarah Seelye, PhD; Hallie C. Prescott, MD, MSc.

**Contents:**

**Supplemental Tables 1a-g**: Broad diagnosis groupings Page 2

**Supplemental Table 2**: Variation across facilities (before dropping facilities with >90% psych hospitalizations) Page 6

**Supplemental Tables 3a-e**: Variation across facilities by tier Page 7

**Supplemental Table 4**: Matching variables by run Page 12

**Supplemental Tables 5a-d**: Variation across matched cohorts for tiers 2-5. Page 13

**Supplemental Figure 1:** Hospital rankings by template matching run 10 versus run 8 Page 18

**Supplemental Figure 2**: Hospital rankings by template matching (run 10) versus regression Page 19

**Supplemental Figure 3**: Hospital rankings by template matching (run 8) versus regression Page 20

**Appendix 1**: Description of VA computing environment Page 21

**Supplemental Tables 1a-g: Diagnostic Code Groupings**

| **Supplemental Table 1a**: Cardiovascular Diagnoses | | | |
| --- | --- | --- | --- |
| **HCUP CCS #** | **HCUP CCS Label** | **N** | **%** |
| 108 | Congestive Heart Failure | 21969 | 3.9% |
| 102 | Nonspecific Chest Pain | 21509 | 3.9% |
| 106 | Cardiac dysrhythmias | 21334 | 3.8% |
| 101 | Coronary atherosclerosis | 19817 | 3.6% |
| 99 | Hypertension with complications | 14930 | 2.7% |
| 100 | Acute myocardial infarction | 8288 | 1.5% |
| 109 | Acute cerebrovascular disease | 6484 | 1.2% |
| 117 | Other circulatory disease | 6125 | 1.1% |
| 103 | Pulmonary heart disease | 3895 | 0.7% |
| 115 | Aortic; peripheral; and visceral artery aneurysms | 3151 | 0.6% |
| 110 | Occlusion or stenosis of precerebral arteries | 2863 | 0.5% |
| 96 | Heart valve disorders | 2765 | 0.5% |
| 112 | Transient cerebral ischemia | 2587 | 0.5% |
| 118 | Phlebitis; thrombophlebitis and thromboembolism | 2525 | 0.5% |

| **Supplemental Table 1b** Psychiatric and Substance Abuse-Related Diagnoses | | | |
| --- | --- | --- | --- |
| **HCUP CCS #** | **HCUP CCS Label** | **N** | **%** |
| 660 | Alcohol-Related Disorders | 33583 | 6.0% |
| 657 | Mood disorders | 26552 | 4.8% |
| 661 | Substance-related disorders | 13765 | 2.5% |
| 659 | Schizophrenia and other psychotic disorders | 12551 | 2.3% |
| 651 | Anxiety disorders | 7976 | 1.4% |
| 662 | Suicide and intentional self-inflicted injury | 2381 | 0.4% |
| 650 | Adjustment disorders | 3233 | 0.6% |

| **Supplemental Table 1c:** Cardiovascular Diagnoses | | | |
| --- | --- | --- | --- |
| **HCUP CCS #** | **HCUP CCS Label** | **N** | **%** |
| 2 | Sepsis | 19233 | 3.5% |
| 122 | Pneumonia | 13493 | 2.4% |
| 197 | Skin and subcutaneous tissue infection | 13359 | 2.4% |
| 159 | Urinary Tract Infection | 9508 | 1.7% |
| 146 | Diverticulosis and diverticulitis | 4534 | 0.8% |
| 135 | Intestinal infection | 4510 | 0.8% |
| 201 | Infective arthritis and osteomyelitis | 4303 | 0.8% |
| 123 | Influenza | 2824 | 0.5% |

| **Supplemental Table 1d**: Gastrointestinal Diagnoses | | | |
| --- | --- | --- | --- |
| **HCUP CCS #** | **HCUP CCS Label** | **N** | **%** |
| 153 | Gastrointestinal hemorrhage | 7841 | 1.4% |
| 149 | Biliary tract disease | 6553 | 1.2% |
| 155 | Other gastrointestinal disorders | 6285 | 1.1% |
| 152 | Pancreatic disorders (not diabetes) | 5970 | 1.1% |
| 143 | Abdominal hernia | 5219 | 0.9% |
| 145 | Intestinal obstruction without hernia | 4407 | 0.8% |
| 58 | Other nutritional; endocrine; and metabolic disorders | 3697 | 0.7% |
| 251 | Abdominal pain | 3593 | 0.6% |
| 151 | Other liver diseases | 3285 | 0.6% |
| 138 | Esophageal disorders | 3069 | 0.6% |
| 16 | Cancer of liver and intrahepatic bile duct | 2924 | 0.5% |
| 154 | Noninfectious gastroenteritis | 2486 | 0.4% |

| **Supplemental Table 1e**: Respiratory Diagnoses | | | |
| --- | --- | --- | --- |
| **HCUP CCS #** | **HCUP CCS Label** | **N** | **%** |
| 127 | Chronic obstructive pulmonary disease and bronchiectasis | 17972 | 3.2% |
| 131 | Respiratory failure; insufficiency; arrest (adult) | 12224 | 2.2% |
| 133 | Other lower respiratory disease | 5838 | 1.0% |
| 19 | Cancer of bronchus; lung | 4832 | 0.9% |
| 129 | Aspiration pneumonitis; food/vomitus | 2838 | 0.5% |
| 130 | Pleurisy; pneumothorax; pulmonary collapse | 2689 | 0.5% |

| **Supplemental Table 1f**: Genitourinary / Renal Diagnoses | | | |
| --- | --- | --- | --- |
| **HCUP CCS #** | **HCUP CCS Label** | **N** | **%** |
| 157 | Acute and unspecified renal failure | 9611 | 1.7% |
| 55 | Fluid and electrolyte disorders | 7224 | 1.3% |
| 164 | Hyperplasia of prostate | 3372 | 0.6% |
| 160 | Calculus of urinary tract | 3221 | 0.6% |
| 29 | Cancer of prostate | 3040 | 0.5% |
| 163 | Genitourinary symptoms and ill-defined conditions | 2888 | 0.5% |
| 161 | Other diseases of kidney and ureters | 2649 | 0.5% |

| **Supplemental Table 1g**: Other Diagnoses | | | |
| --- | --- | --- | --- |
| **HCUP CCS #** | **HCUP CCS Label** | **N** | **%** |
| 203 | Osteoarthritis | 13242 | 2.4% |
| 237 | Complication of device; implant or graft | 11254 | 2.0% |
| 50 | Diabetes mellitus with complications | 10908 | 2.0% |
| 238 | Complications of surgical procedures or medical care | 10295 | 1.9% |
| 205 | Spondylosis; intervertebral disc disorders; other back problems | 9076 | 1.6% |
| 95 | Other nervous system disorders | 7913 | 1.4% |
| 245 | Syncope | 6640 | 1.2% |
| 59 | Deficiency and other anemia | 5807 | 1.0% |
| 653 | Delirium dementia and amnestic and other cognitive disorders | 5760 | 1.0% |
| 211 | Other connective tissue disease | 4645 | 0.8% |
| 259 | Residual codes; unclassified | 4533 | 0.8% |
| 257 | Other aftercare | 3222 | 0.6% |
| 83 | Epilepsy; convulsions | 2945 | 0.5% |
| 42 | Secondary malignancies | 2845 | 0.5% |
| 47 | Other and unspecified benign neoplasm | 2832 | 0.5% |
| 45 | Maintenance chemotherapy; radiotherapy | 2742 | 0.5% |
| 252 | Malaise and fatigue | 2597 | 0.5% |

| **Supplemental Table 2**: Case-mix variation across VA facilities before removal of psychiatric hospitals | | | | | | | | | |
| --- | --- | --- | --- | --- | --- | --- | --- | --- | --- |
|  | Range across Facilities | | | | | | | Test Statistic* | *p** |
|  | min | 12.5^th^ percentile | 25^th^ percentile | median | 75^th^ percentile | 87.5^th^ percentile | max |  |  |
| Predicted mortality, median % | 0.2 | 1.1 | 1.4 | 1.6 | 1.9 | 2.1 | 3.6 | 29,125 | <0.001 |
| Surgical Operations, % | 0.0 | 0.5 | 5.0 | 8.7 | 11.7 | 13.3 | 21.0 | 11,554 | <0.001 |
| Demographics |  |  |  |  |  |  |  |  |  |
| Age in years, median | 47 | 63 | 65 | 67 | 68 | 69 | 75 | 23,955 | <0.001 |
| Female, % | 2.3 | 4.2 | 4.8 | 5.6 | 6.5 | 7.6 | 15.7 | 2,622 | <0.001 |
| Black, % | 0.4 | 3.1 | 6.4 | 14.8 | 32.1 | 44.1 | 70.8 | 89,905 | <0.001 |
| White, % | 23.0 | 49.7 | 59.9 | 75.7 | 86.6 | 90.2 | 95.1 | 70,624 | <0.001 |
| Hispanic, % | 0.2 | 0.6 | 1.1 | 1.8 | 5.1 | 10.4 | 93.3 | 148,788 | <0.001 |
| Comorbidities, % |  |  |  |  |  |  |  |  |  |
| Depression | 4.0 | 11.4 | 13.6 | 17.1 | 20.0 | 22.8 | 33.6 | 8,174 | <0.001 |
| Liver disease | 1.4 | 4.2 | 5.1 | 6.3 | 7.9 | 8.9 | 19.3 | 5,226 | <0.001 |
| Metastatic cancer | 0.0 | 0.9 | 1.5 | 1.9 | 2.4 | 2.7 | 3.7 | 996 | <0.001 |
| Paralysis | 0.3 | 1.4 | 1.7 | 2.3 | 3.0 | 3.6 | 4.8 | 1,676 | <0.001 |
| Pulmonary disease | 10.0 | 16.9 | 18.3 | 22.0 | 26.0 | 27.8 | 36.8 | 6,333 | <0.001 |
| Renal disease | 1.3 | 11.6 | 14.3 | 17.0 | 19.9 | 21.4 | 25.3 | 5,239 | <0.001 |
| Admission source, % |  |  |  |  |  |  |  |  |  |
| Nursing home | 0.0 | 0.5 | 0.9 | 1.7 | 2.6 | 3.5 | 7.3 | 4,836 | <0.001 |
| Emergency dept. | 0.1 | 4.0 | 8.5 | 29.5 | 68.9 | 84.7 | 98.7 | 251,420 | <0.001 |
| Adm diagnosis group, % |  |  |  |  |  |  |  |  |  |
| Cardiovascular | 0.0 | 16.5 | 20.7 | 25.1 | 28.1 | 30.3 | 38.2 | 8,784 | <0.001 |
| Psych / substance | 2.7 | 10.8 | 13.1 | 16.9 | 22.0 | 33.7 | 99.8 | 58,879 | <0.001 |
| Infection | 0.0 | 9.6 | 11.0 | 13.1 | 15.0 | 16.8 | 23.8 | 4,875 | <0.001 |
| Gastrointestinal | 0.0 | 7.4 | 9.0 | 9.9 | 10.9 | 11.9 | 15.8 | 2,235 | <0.001 |
| Respiratory | 0.0 | 6.2 | 7.3 | 8.2 | 9.9 | 11.4 | 16.8 | 3,139 | <0.001 |
| GU/renal | 0.0 | 3.7 | 4.6 | 5.6 | 6.5 | 7.1 | 9.4 | 2,067 | <0.001 |
| Other | 0.2 | 12.2 | 16.1 | 18.9 | 21.0 | 23.1 | 26.9 | 5,655 | <0.001 |
| *Based on the Kruskal-Wallis test for continuous variables and Pearson’s Chi-Squared test for categorical variables | | | | | | | | | |

| **Supplemental Table 3a**: Case-mix variation across VA facilities (after removal of psych hospitals), Tier 1 | | | | | | | | | |
| --- | --- | --- | --- | --- | --- | --- | --- | --- | --- |
|  | Range across Facilities | | | | | | | Test Statistic* | *p** |
|  | min | 12.5^th^ percentile | 25^th^ percentile | median | 75^th^ percentile | 87.5^th^ percentile | max |  |  |
| Predicted mortality, median | 0.2 | 1.5 | 1.6 | 1.8 | 2.0 | 2.2 | 3.2 | 16,025 | <0.001 |
| Surgical Operations, % | 0.0 | 4.9 | 8.0 | 9.8 | 11.9 | 15.0 | 20.2 | 13,438 | <0.001 |
| Demographics |  |  |  |  |  |  |  |  |  |
| Age in years, median | 52 | 65 | 65 | 67 | 68 | 68 | 75 | 13,438 | <0.001 |
| Female, % | 2.3 | 4.3 | 4.8 | 5.5 | 6.3 | 6.9 | 9.7 | 1,007 | <0.001 |
| Black, % | 3.0 | 9.0 | 13.3 | 20.0 | 37.7 | 50.3 | 70.8 | 54,693 | <0.001 |
| White, % | 23.0 | 47.2 | 55.2 | 71.2 | 78.7 | 85.5 | 92.7 | 42,869 | <0.001 |
| Hispanic, % | 0.4 | 0.9 | 1.2 | 1.8 | 5.6 | 14.1 | 93.3 | 118,984 | <0.001 |
| Comorbidities, % |  |  |  |  |  |  |  |  |  |
| Depression | 9.7 | 11.8 | 15.0 | 17.6 | 19.6 | 22.3 | 33.5 | 4,218 | <0.001 |
| Liver disease | 2.6 | 4.9 | 5.9 | 7.4 | 8.8 | 11.0 | 19.3 | 3,461 | <0.001 |
| Metastatic cancer | 0.0 | 1.6 | 1.8 | 2.2 | 2.5 | 2.7 | 3.7 | 455 | <0.001 |
| Paralysis | 0.3 | 1.7 | 2.1 | 2.6 | 3.4 | 3.9 | 4.8 | 977 | <0.001 |
| Pulmonary disease | 10.0 | 16.5 | 18.2 | 20.8 | 24.6 | 26.1 | 36.8 | 3,675 | <0.001 |
| Renal disease | 1.4 | 13.2 | 16.2 | 18.6 | 20.4 | 21.3 | 25.3 | 2,721 | <0.001 |
| Admission source, % |  |  |  |  |  |  |  |  |  |
| Nursing home | 0.0 | 0.4 | 0.7 | 1.5 | 2.1 | 2.8 | 7.3 | 2,829 | <0.001 |
| Emergency dept. | 0.2 | 10.8 | 18.6 | 52.7 | 81.7 | 90.8 | 98.3 | 162,296 | <0.001 |
| Adm diagnosis group, % |  |  |  |  |  |  |  |  |  |
| Cardiovascular | 0.0 | 22.8 | 24.6 | 27.0 | 29.4 | 31.1 | 38.2 | 4,094 | <0.001 |
| Psych / substance | 2.8 | 9.9 | 13.0 | 15.3 | 19.0 | 21.3 | 98.8 | 33,510 | <0.001 |
| Infection | 0.0 | 9.8 | 10.5 | 12.4 | 14.1 | 15.3 | 19.6 | 2,584 | <0.001 |
| Gastrointestinal | 0.0 | 8.8 | 9.3 | 10.0 | 10.8 | 11.8 | 13.0 | 1,160 | <0.001 |
| Respiratory | 0.0 | 6.1 | 6.9 | 7.7 | 8.7 | 10.0 | 13.4 | 1,540 | <0.001 |
| GU/renal | 0.0 | 4.7 | 5.3 | 6.0 | 6.5 | 6.9 | 9.3 | 1,065 | <0.001 |
| Other | 0.8 | 16.1 | 18.4 | 19.9 | 21.6 | 24.0 | 26.9 | 2,819 | <0.001 |
| *Based on the Kruskal-Wallis test for continuous variables and Pearson’s Chi-Squared test for categorical variables | | | | | | | | | |

| **Supplemental Table 3b**: Case-mix variation across VA facilities (after removal of psych hospitals), Tier 2 | | | | | | | | | |
| --- | --- | --- | --- | --- | --- | --- | --- | --- | --- |
|  | Range across Facilities | | | | | | | Test Statistic* | *p** |
|  | min | 12.5^th^ percentile | 25^th^ percentile | median | 75^th^ percentile | 87.5^th^ percentile | max |  |  |
| Predicted mortality, median | 0.2 | 1.5 | 1.6 | 1.8 | 2.1 | 2.2 | 2.4 | 4,297 | <0.001 |
| Surgical Operations, % | 0.0 | 5.1 | 7.0 | 8.7 | 12.1 | 12.7 | 14.1 | 2,598 | <0.001 |
| Demographics |  |  |  |  |  |  |  |  |  |
| Age in years, median | 47 | 66 | 66 | 67 | 68 | 68 | 69 | 2,598 | <0.001 |
| Female, % | 3.6 | 4.3 | 4.7 | 5.8 | 6.1 | 6.5 | 13.3 | 295 | <0.001 |
| Black, % | 2.3 | 7.3 | 9.5 | 13.8 | 19.6 | 39.8 | 54.5 | 10,866 | <0.001 |
| White, % | 40.5 | 54.0 | 61.2 | 77.9 | 87.1 | 89.5 | 93.2 | 9,830 | <0.001 |
| Hispanic, % | 0.2 | 0.5 | 0.8 | 1.9 | 6.5 | 9.6 | 24.7 | 6,240 | <0.001 |
| Comorbidities, % |  |  |  |  |  |  |  |  |  |
| Depression | 8.5 | 10.4 | 14.3 | 15.9 | 18.3 | 20.1 | 24.1 | 1,262 | <0.001 |
| Liver disease | 3.3 | 3.9 | 5.2 | 5.5 | 7.4 | 8.3 | 9.8 | 449 | <0.001 |
| Metastatic cancer | 0.1 | 1.3 | 1.5 | 1.8 | 2.1 | 2.7 | 2.9 | 150 | <0.001 |
| Paralysis | 0.4 | 1.7 | 1.9 | 2.2 | 2.8 | 3.2 | 4.0 | 181 | <0.001 |
| Pulmonary disease | 10.9 | 16.3 | 18.9 | 24.5 | 27.2 | 28.2 | 30.0 | 1,201 | <0.001 |
| Renal disease | 1.3 | 14.1 | 14.7 | 17.1 | 19.0 | 19.5 | 22.4 | 645 | <0.001 |
| Admission source, % |  |  |  |  |  |  |  |  |  |
| Nursing home | 0.0 | 0.6 | 0.9 | 1.5 | 3.0 | 3.6 | 5.5 | 828 | <0.001 |
| Emergency dept. | 0.5 | 9.0 | 21.1 | 39.7 | 80.6 | 85.1 | 98.7 | 36,473 | <0.001 |
| Adm diagnosis group, % |  |  |  |  |  |  |  |  |  |
| Cardiovascular | 0.0 | 18.1 | 22.1 | 25.3 | 27.4 | 29.8 | 34.8 | 1,280 | <0.001 |
| Psych / substance | 2.7 | 12.1 | 14.8 | 17.6 | 20.5 | 24.5 | 99.0 | 8,163 | <0.001 |
| Infection | 0.0 | 9.6 | 10.7 | 12.1 | 15.0 | 16.0 | 21.5 | 853 | <0.001 |
| Gastrointestinal | 0.0 | 9.0 | 9.1 | 9.7 | 11.2 | 11.6 | 12.3 | 287 | <0.001 |
| Respiratory | 0.1 | 7.0 | 7.6 | 9.3 | 10.3 | 10.9 | 11.8 | 435 | <0.001 |
| GU/renal | 0.0 | 4.3 | 5.4 | 6.0 | 7.1 | 7.5 | 8.5 | 300 | <0.001 |
| Other | 0.9 | 16.1 | 17.7 | 19.0 | 20.5 | 21.6 | 23.7 | 573 | <0.001 |
| *Based on the Kruskal-Wallis test for continuous variables and Pearson’s Chi-Squared test for categorical variables | | | | | | | | | |

| **Supplemental Table 3c**: Case-mix variation across VA facilities (after removal of psych hospitals), Tier 3 | | | | | | | | | |
| --- | --- | --- | --- | --- | --- | --- | --- | --- | --- |
|  | Range across Facilities | | | | | | | Test Statistic* | *p** |
|  | min | 12.5^th^ percentile | 25^th^ percentile | median | 75^th^ percentile | 87.5^th^ percentile | max |  |  |
| Predicted mortality, median | 0.6 | 1.3 | 1.7 | 2.0 | 2.3 | 2.6 | 3.0 | 3,316 | <0.001 |
| Surgical Operations, % | 0.5 | 2.0 | 4.9 | 7.9 | 11.1 | 12.4 | 21.0 | 4,022 | <0.001 |
| Demographics |  |  |  |  |  |  |  |  |  |
| Age in years, median | 57 | 64 | 67 | 68 | 69 | 69 | 70 | 4,022 | <0.001 |
| Female, % | 3.4 | 4.2 | 4.9 | 5.5 | 7.0 | 8.3 | 15.7 | 986 | <0.001 |
| Black, % | 0.4 | 1.0 | 2.2 | 5.1 | 14.6 | 24.4 | 58.3 | 12,132 | <0.001 |
| White, % | 37.9 | 67.2 | 74.2 | 87.2 | 90.4 | 93.0 | 95.1 | 7,950 | <0.001 |
| Hispanic, % | 0.3 | 0.6 | 1.1 | 2.0 | 3.8 | 6.1 | 20.1 | 6,095 | <0.001 |
| Comorbidities, % |  |  |  |  |  |  |  |  |  |
| Depression | 7.2 | 9.0 | 12.5 | 15.1 | 19.7 | 22.9 | 33.6 | 1,636 | <0.001 |
| Liver disease | 2.2 | 3.7 | 4.6 | 5.7 | 7.0 | 7.9 | 8.7 | 445 | <0.001 |
| Metastatic cancer | 0.3 | 0.8 | 1.3 | 1.8 | 2.2 | 2.5 | 3.1 | 171 | <0.001 |
| Paralysis | 0.5 | 1.2 | 1.5 | 2.1 | 2.4 | 2.6 | 4.4 | 219 | <0.001 |
| Pulmonary disease | 15.2 | 18.0 | 19.8 | 22.9 | 27.3 | 29.9 | 35.3 | 923 | <0.001 |
| Renal disease | 8.7 | 11.8 | 13.5 | 15.2 | 17.3 | 21.4 | 23.9 | 688 | <0.001 |
| Admission source, % |  |  |  |  |  |  |  |  |  |
| Nursing home | 0.0 | 0.5 | 1.0 | 1.9 | 2.8 | 3.6 | 6.3 | 821 | <0.001 |
| Emergency dept. | 0.1 | 1.0 | 2.3 | 8.3 | 31.8 | 47.9 | 76.4 | 27,589 | <0.001 |
| Adm diagnosis group, % |  |  |  |  |  |  |  |  |  |
| Cardiovascular | 11.6 | 15.3 | 18.7 | 20.7 | 24.4 | 26.6 | 33.1 | 1,200 | <0.001 |
| Psych / substance | 3.7 | 12.2 | 14.5 | 19.7 | 25.6 | 30.6 | 56.0 | 4,630 | <0.001 |
| Infection | 8.9 | 12.5 | 13.2 | 14.3 | 15.5 | 18.2 | 23.8 | 641 | <0.001 |
| Gastrointestinal | 4.1 | 7.5 | 8.6 | 10.4 | 11.5 | 12.5 | 15.8 | 385 | <0.001 |
| Respiratory | 5.4 | 7.2 | 7.8 | 9.1 | 10.5 | 11.8 | 16.0 | 427 | <0.001 |
| GU/renal | 2.3 | 3.6 | 4.5 | 5.2 | 6.1 | 6.9 | 9.4 | 409 | <0.001 |
| Other | 7.7 | 11.6 | 14.1 | 17.5 | 20.0 | 22.5 | 26.0 | 913 | <0.001 |
| *Based on the Kruskal-Wallis test for continuous variables and Pearson’s Chi-Squared test for categorical variables | | | | | | | | | |

| **Supplemental Table 3d**: Case-mix variation across VA facilities (after removal of psych hospitals), Tier 4 | | | | | | | | | |
| --- | --- | --- | --- | --- | --- | --- | --- | --- | --- |
|  | Range across Facilities | | | | | | | Test Statistic* | *p** |
|  | min | 12.5^th^ percentile | 25^th^ percentile | median | 75^th^ percentile | 87.5^th^ percentile | max |  |  |
| Predicted mortality, median % | 0.9 | 1.5 | 2.1 | 2.4 | 3.0 | 3.0 | 3.0 | 911 | <0.001 |
| Surgical Operations, % | 0.0 | 0.0 | 0.1 | 0.6 | 1.5 | 1.5 | 1.6 | 720 | <0.001 |
| Demographics |  |  |  |  |  |  |  |  | <0.001 |
| Age in years, median | 60 | 62 | 64 | 67 | 68 | 70 | 71 | 720 | <0.001 |
| Female, % | 2.5 | 3.3 | 4.1 | 7.8 | 8.1 | 9.6 | 11.1 | 127 | <0.001 |
| Black, % | 2.7 | 4.6 | 6.4 | 8.1 | 49.4 | 53.5 | 57.5 | 2,058 | <0.001 |
| White, % | 39.1 | 40.9 | 42.7 | 86.4 | 87.3 | 88.9 | 90.5 | 1,818 | <0.001 |
| Hispanic, % | 0.2 | 0.4 | 0.6 | 1.7 | 2.0 | 2.5 | 3.1 | 57 | <0.001 |
| Comorbidities, % |  |  |  |  |  |  |  |  |  |
| Depression | 4.0 | 8.6 | 13.2 | 19.4 | 23.7 | 24.4 | 25.1 | 353 | <0.001 |
| Liver disease | 4.2 | 4.5 | 4.8 | 5.5 | 5.6 | 5.9 | 6.1 | 7 | 0.132 |
| Metastatic cancer | 0.3 | 0.6 | 0.9 | 1.7 | 1.8 | 2.3 | 2.8 | 36 | <0.001 |
| Paralysis | 1.5 | 1.6 | 1.7 | 1.9 | 2.3 | 2.9 | 3.6 | 16 | 0.004 |
| Pulmonary disease | 18.2 | 20.1 | 21.9 | 27.1 | 27.3 | 27.4 | 27.5 | 63 | <0.001 |
| Renal disease | 11.4 | 11.5 | 11.6 | 21.0 | 21.8 | 22.8 | 23.9 | 159 | <0.001 |
| Admission source, % |  |  |  |  |  |  |  |  |  |
| Nursing home | 0.4 | 1.3 | 2.2 | 2.6 | 2.9 | 3.2 | 3.5 | 44 | <0.001 |
| Emergency dept. | 0.6 | 2.5 | 4.5 | 5.3 | 18.3 | 27.7 | 37.2 | 1,153 | <0.001 |
| Adm diagnosis group, % |  |  |  |  |  |  |  |  |  |
| Cardiovascular | 19.2 | 20.0 | 20.8 | 27.0 | 29.0 | 30.1 | 31.2 | 72 | <0.001 |
| Psych / substance | 4.4 | 6.8 | 9.3 | 12.4 | 24.7 | 29.5 | 34.3 | 696 | <0.001 |
| Infection | 9.4 | 11.9 | 14.3 | 15.3 | 16.0 | 18.2 | 20.4 | 93 | <0.001 |
| Gastrointestinal | 6.4 | 7.0 | 7.6 | 8.4 | 10.1 | 10.4 | 10.7 | 29 | <0.001 |
| Respiratory | 8.2 | 10.0 | 11.7 | 12.8 | 14.6 | 15.7 | 16.8 | 69 | <0.001 |
| GU/renal | 3.3 | 3.7 | 4.1 | 4.5 | 5.6 | 6.6 | 7.6 | 35 | <0.001 |
| Other | 9.3 | 11.6 | 13.9 | 16.2 | 19.3 | 20.2 | 21.2 | 102 | <0.001 |
| *Based on the Kruskal-Wallis test for continuous variables and Pearson’s Chi-Squared test for categorical variables | | | | | | | | | |

| **Supplemental Table 3e**: Case-mix variation across VA facilities (after removal of psych hospitals), Tier 5 | | | | | | | | | |
| --- | --- | --- | --- | --- | --- | --- | --- | --- | --- |
|  | Range across Facilities | | | | | | | Test Statistic* | *p** |
|  | min | 12.5^th^ percentile | 25^th^ percentile | median | 75^th^ percentile | 87.5^th^ percentile | max |  |  |
| Predicted mortality, median % | 0.2 | 0.3 | 0.5 | 1.3 | 1.3 | 1.7 | 2.6 | 2,746 | <0.001 |
| Surgical Operations, % | 0.0 | 0.0 | 0.0 | 0.0 | 0.0 | 0.1 | 0.2 | 1,415 | 0.132 |
| Demographics |  |  |  |  |  |  |  |  |  |
| Age in years, median | 53 | 56 | 57 | 62 | 63 | 65 | 70 | 1,415 | <0.001 |
| Female, % | 4.6 | 5.1 | 5.9 | 7.2 | 7.4 | 7.8 | 8.3 | 21 | 0.002 |
| Black, % | 1.1 | 2.1 | 5.8 | 13.5 | 25.8 | 38.6 | 43.6 | 1,543 | <0.001 |
| White, % | 50.3 | 57.5 | 69.6 | 80.8 | 85.2 | 89.8 | 93.2 | 1,002 | <0.001 |
| Hispanic, % | 0.2 | 0.8 | 1.1 | 1.3 | 2.2 | 2.6 | 3.6 | 69 | <0.001 |
| Comorbidities, % |  |  |  |  |  |  |  |  |  |
| Depression | 5.4 | 11.6 | 15.8 | 22.0 | 24.4 | 27.8 | 33.6 | 395 | <0.001 |
| Liver disease | 1.4 | 2.2 | 2.5 | 5.4 | 5.7 | 6.2 | 7.0 | 89 | <0.001 |
| Metastatic cancer | 0.0 | 0.5 | 0.7 | 0.7 | 0.8 | 0.9 | 0.9 | 11 | 0.065 |
| Paralysis | 0.3 | 0.6 | 0.7 | 1.3 | 1.5 | 1.9 | 2.5 | 38 | <0.001 |
| Pulmonary disease | 10.5 | 13.3 | 15.4 | 18.0 | 25.9 | 27.3 | 27.8 | 238 | <0.001 |
| Renal disease | 1.3 | 4.2 | 5.2 | 7.9 | 11.4 | 14.3 | 16.5 | 280 | <0.001 |
| Admission source, % |  |  |  |  |  |  |  |  |  |
| Nursing home | 0.7 | 1.2 | 1.5 | 1.7 | 2.9 | 3.9 | 5.4 | 105 | <0.001 |
| Emergency dept. | 4.5 | 4.8 | 5.2 | 7.5 | 12.0 | 24.2 | 58.8 | 2,332 | <0.001 |
| Adm diagnosis group, % |  |  |  |  |  |  |  |  |  |
| Cardiovascular | 0.0 | 4.1 | 6.1 | 12.6 | 13.3 | 16.4 | 24.9 | 507 | <0.001 |
| Psych / substance | 8.7 | 32.6 | 41.5 | 51.8 | 69.3 | 78.2 | 99.8 | 2,690 | <0.001 |
| Infection | 0.0 | 4.7 | 6.8 | 9.6 | 13.2 | 18.1 | 22.0 | 446 | <0.001 |
| Gastrointestinal | 0.0 | 2.5 | 3.5 | 4.7 | 6.2 | 7.4 | 9.4 | 148 | <0.001 |
| Respiratory | 0.0 | 2.4 | 3.9 | 7.4 | 9.0 | 11.1 | 12.8 | 246 | <0.001 |
| GU/renal | 0.1 | 1.8 | 2.4 | 3.1 | 3.8 | 4.8 | 6.9 | 113 | <0.001 |
| Other | 0.2 | 4.4 | 7.3 | 9.2 | 13.7 | 15.7 | 17.1 | 311 | <0.001 |
| *Based on the Kruskal-Wallis test for continuous variables and Pearson’s Chi-Squared test for categorical variables | | | | | | | | | |

| **Supplemental Table 4**: Matching Algorithm Variable lists | | | | | | | | | | | | |
| --- | --- | --- | --- | --- | --- | --- | --- | --- | --- | --- | --- | --- |
| Variable | Type | N (cum) | Matching algorithm | | | | | | | | | |
|  |  |  | 1 | 2 | 3 | 4 | 5 | 6 | 7 | 8 | 9 | 10 |
| Predicted Mortality | Continuous | 1 (1) | ✓ | ✓ | ✓ | ✓ | ✓ | ✓ | ✓ | ✓ | ✓ | ✓ |
| Predicted mortality quintile (deciles for run 3) | Categorical | 1 (2) | ✓ | ✓ | ✓ | ✓ | ✓ | ✓ | ✓ | ✓ | ✓ | ✓ |
| Age | Continuous | 1 (3) | ✓ | ✓ | ✓ | ✓ | ✓ | ✓ | ✓ | ✓ | ✓ | ✓ |
| Sex | Indicator | 1 (4) | ✓ | ✓ | ✓ | ✓ | ✓ | ✓ | ✓ | ✓ | ✓ | ✓ |
| Race/ethnicity (black, white, Hispanic) | Indicators | 3 (7) | ✓ | ✓ | ✓ | ✓ | ✓ | ✓ | ✓ | ✓ |  |  |
| Admission source (nursing facility, ER) | Indicators | 2 (9) | ✓ | ✓ | ✓ | ✓ | ✓ | ✓ | ✓ | ✓ | ✓ | ✓ |
| Comorbidities (CHF, chronic pulmonary disease, paralysis, renal failure, liver disease, metastatic cancer, depression) | Indicator | 7 (16) | ✓ | ✓ | ✓ | ✓ | ✓ | ✓ | ✓ | ✓ | ✓ | ✓ |
| Additional comorbidities (valvular disease, pulmonary circulation disorders, peripheral vascular disorders, hypertension, other neurological disorders, uncomplicated diabetes, complicated diabetes, hypothyroidism, peptic ulcer disease (excluding bleeding), AIDS, lymphoma, solid tumor without metastasis, rheumatoid arthritis/collagen vascular diseases, coagulopathy, obesity, weight loss, fluid and electrolyte disorders, blood loss anemia, deficiency anemias, alcohol abuse, drug abuse, psychoses | Indicators | 22 (38) | ✓ | ✓ | ✓ |  |  |  |  |  |  |  |
| Surgical indicator (major surgery within 24 hours of admission) | Indicator | 1 (39) | ✓ | ✓ | ✓ | ✓ | ✓ | ✓ | ✓ | ✓ | ✓ | ✓ |
| Laboratories drawn within 24 hours of admission (albumin, bilirubin, gfr, BUN, sodium, glucose, hematocrit, pao2, pco2, pH, white blood cell count) | Continuous | 11 (50) | ✓ | ✓ | ✓ | ✓ | ✓ | ✓ | ✓ | ✓ |  |  |
| Broad principal diagnosis category (cardiovascular, psychiatric/substance abuse, infection, gastrointestinal disease, genitourinary disease, respiratory disease, other) | Indicators | 7 (57) |  |  |  | ✓ | ✓ | ✓ | ✓ | ✓ | ✓ | ✓ |
| Narrow principal diagnosis category (There are 72 Healthcare Cost and Utilization Project (HCUP) principal diagnosis categories in the dataset (after excluding diagnosis categories that occur in <1/300 hospitalizations) and other patient-level data not included elsewhere | Categorical (treated as indicators) | 72 (129) | ✓ |  |  |  |  |  |  |  |  |  |

| **Supplemental Table 5a**: Case-mix variation across matched cohorts for 20 Tier 2 hospitals, using matching run 10 | | | | | | | | | |
| --- | --- | --- | --- | --- | --- | --- | --- | --- | --- |
|  | Range across Facilities | | | | | | | Test Statistic* | *p** |
|  | min | 12.5^th^ percentile | 25^th^ percentile | median | 75^th^ percentile | 87.5^th^ percentile | max |  |  |
| Predicted probability of mortality, median % | 1.5 | 1.5 | 1.6 | 1.6 | 1.6 | 1.7 | 1.8 | 1 | >0.999 |
| Surgical Operations, % | 8.7 | 8.7 | 8.7 | 8.7 | 8.7 | 8.7 | 8.7 |  | >0.999 |
| Demographics |  |  |  |  |  |  |  |  |  |
| Age in years, median | 66 | 67 | 67 | 67 | 67 | 67 | 68 | 3 | >0.999 |
| Female, % | 3.7 | 4.0 | 4.0 | 4.3 | 4.7 | 5.2 | 5.3 | 4 | >0.999 |
| Black, % | 3.7 | 6.1 | 8.8 | 13.0 | 17.8 | 37.8 | 50.3 | 760 | <0.001 |
| White, % | 46.7 | 56.0 | 69.7 | 82.0 | 87.0 | 90.8 | 92.0 | 662 | <0.001 |
| Hispanic, % | 0.3 | 0.3 | 1.0 | 2.0 | 4.8 | 8.2 | 25.3 | 519 | <0.001 |
| Comorbidities, % |  |  |  |  |  |  |  |  |  |
| Depression | 9.7 | 11.1 | 11.3 | 12.0 | 12.4 | 13.0 | 13.7 | 5 | >0.999 |
| Liver disease | 4.0 | 4.0 | 4.3 | 5.0 | 5.4 | 5.7 | 6.0 | 6 | >0.999 |
| Metastatic cancer | 0.7 | 0.7 | 0.9 | 1.3 | 2.0 | 2.0 | 2.0 | 13 | 0.858 |
| Paralysis | 0.7 | 0.7 | 0.7 | 1.3 | 1.7 | 1.7 | 2.3 | 12 | 0.909 |
| Pulmonary disease | 20.0 | 20.8 | 21.5 | 22.5 | 22.7 | 23.0 | 23.3 | 3 | >0.999 |
| Renal disease | 15.0 | 15.5 | 16.0 | 16.3 | 16.7 | 16.9 | 17.3 | 1 | >0.999 |
| Admission source, % |  |  |  |  |  |  |  |  |  |
| Nursing home | 0.0 | 0.3 | 0.6 | 1.2 | 1.7 | 1.9 | 2.3 | 23 | 0.234 |
| Emergency dept. | 5.7 | 32.1 | 40.2 | 43.7 | 44.9 | 55.5 | 89.7 | 580 | <0.001 |
| Broad diagnosis group, % |  |  |  |  |  |  |  |  |  |
| Cardiovascular | 24.3 | 24.3 | 24.3 | 24.3 | 24.3 | 24.3 | 24.3 | 0 | >0.999 |
| Psych / substance | 17.0 | 17.0 | 17.0 | 17.0 | 17.0 | 17.0 | 17.0 | 0 | >0.999 |
| Infection | 14.0 | 14.0 | 14.0 | 14.0 | 14.0 | 14.0 | 14.0 | 0 | >0.999 |
| Gastrointestinal | 9.0 | 9.0 | 9.0 | 9.0 | 9.0 | 9.0 | 9.0 | 0 | >0.999 |
| Respiratory | 9.7 | 9.7 | 9.7 | 9.7 | 9.7 | 9.7 | 9.7 | 0 | >0.999 |
| GU/renal | 4.7 | 4.7 | 4.7 | 4.7 | 4.7 | 4.7 | 4.7 | 0 | >0.999 |
| Other | 21.3 | 21.3 | 21.3 | 21.3 | 21.3 | 21.3 | 21.3 | 0 | >0.999 |
| *Based on the Kruskal-Wallis test for continuous variables and Pearson’s Chi-Squared test for categorical variables | | | | | | | | | |

| **Supplemental Table 5b**: Case-mix variation across matched cohorts for 31 Tier 3 hospitals, using matching run 10 | | | | | | | | | |
| --- | --- | --- | --- | --- | --- | --- | --- | --- | --- |
|  | Range across Facilities | | | | | | | Test Statistic* | *p** |
|  | min | 12.5^th^ percentile | 25^th^ percentile | median | 75^th^ percentile | 87.5^th^ percentile | max |  |  |
| Predicted probability of mortality, median % | 1.8 | 1.9 | 1.9 | 2.0 | 2.0 | 2.0 | 2.1 | 4 | >0.999 |
| Surgical Operations, % | 7.7 | 7.7 | 7.7 | 7.7 | 7.7 | 7.7 | 7.7 |  | >0.999 |
| Demographics |  |  |  |  |  |  |  |  |  |
| Age in years, median | 66 | 67 | 67 | 67 | 67 | 68 | 68 | 6 | >0.999 |
| Female, % | 3.7 | 4.3 | 4.3 | 4.7 | 5.2 | 5.6 | 5.7 | 5 | >0.999 |
| Black, % | 0.3 | 1.3 | 2.2 | 5.3 | 11.5 | 19.0 | 57.0 | 1,349 | <0.001 |
| White, % | 40.0 | 70.8 | 76.2 | 86.0 | 90.7 | 93.5 | 95.7 | 880 | <0.001 |
| Hispanic, % | 0.0 | 0.3 | 0.7 | 2.0 | 5.0 | 8.3 | 17.3 | 479 | <0.001 |
| Comorbidities, % |  |  |  |  |  |  |  |  |  |
| Depression | 8.3 | 12.5 | 13.7 | 14.3 | 15.2 | 15.9 | 17.7 | 23 | 0.635 |
| Liver disease | 2.7 | 3.5 | 4.3 | 4.7 | 5.2 | 5.6 | 6.0 | 13 | 0.985 |
| Metastatic cancer | 0.0 | 0.4 | 0.7 | 1.0 | 1.3 | 1.3 | 1.7 | 15 | 0.971 |
| Paralysis | 0.0 | 0.4 | 0.7 | 1.0 | 1.2 | 1.3 | 2.0 | 17 | 0.917 |
| Pulmonary disease | 22.3 | 23.3 | 23.7 | 24.0 | 24.7 | 25.3 | 26.3 | 4 | >0.999 |
| Renal disease | 11.7 | 14.3 | 14.7 | 15.0 | 15.7 | 15.9 | 16.3 | 7 | >0.999 |
| Admission source, % |  |  |  |  |  |  |  |  |  |
| Nursing home | 0.0 | 0.4 | 1.0 | 1.7 | 2.0 | 2.0 | 2.7 | 31 | 0.252 |
| Emergency dept. | 0.3 | 7.0 | 10.0 | 17.7 | 21.7 | 26.2 | 42.7 | 588 | <0.001 |
| Broad diagnosis group, % |  |  |  |  |  |  |  |  |  |
| Cardiovascular | 20.7 | 21.3 | 21.7 | 21.7 | 21.7 | 21.7 | 23.3 | 1 | >0.999 |
| Psych / substance | 17.3 | 21.7 | 21.7 | 21.7 | 21.7 | 21.7 | 21.7 | 3 | >0.999 |
| Infection | 14.3 | 14.3 | 14.3 | 14.3 | 14.3 | 14.6 | 15.0 | 0 | >0.999 |
| Gastrointestinal | 10.7 | 11.0 | 11.0 | 11.0 | 11.0 | 11.6 | 12.0 | 1 | >0.999 |
| Respiratory | 8.0 | 8.0 | 8.0 | 8.0 | 8.0 | 8.0 | 8.3 | 0 | >0.999 |
| GU/renal | 4.0 | 4.0 | 5.0 | 5.0 | 5.0 | 5.0 | 5.3 | 3 | >0.999 |
| Other | 18.3 | 18.3 | 18.3 | 18.3 | 18.3 | 18.7 | 20.0 | 1 | >0.999 |
| *Based on the Kruskal-Wallis test for continuous variables and Pearson’s Chi-Squared test for categorical variables | | | | | | | | | |

| **Supplemental Table 5c**: Case-mix variation across matched cohorts for 5 Tier 4 hospitals, using matching run 10 | | | | | | | | | |
| --- | --- | --- | --- | --- | --- | --- | --- | --- | --- |
|  | Range across Facilities | | | | | | | Test Statistic* | *p** |
|  | min | 12.5^th^ percentile | 25^th^ percentile | median | 75^th^ percentile | 87.5^th^ percentile | max |  |  |
| Predicted probability of mortality, median % | 1.8 | 1.8 | 1.8 | 1.8 | 1.9 | 2.0 | 2.1 | 1 | 0.965 |
| Surgical Operations, % | 0.0 | 0.0 | 0.0 | 0.0 | 0.0 | 0.0 | 0.0 |  | >0.999 |
| Demographics |  |  |  |  |  |  |  |  |  |
| Age in years, median | 65 | 66 | 66 | 67 | 67 | 67 | 67 | 2 | 0.801 |
| Female, % | 3.3 | 4.2 | 5.0 | 5.7 | 6.0 | 6.5 | 7.0 | 4 | 0.369 |
| Black, % | 2.7 | 5.3 | 8.0 | 9.7 | 42.7 | 48.3 | 54.0 | 362 | <0.001 |
| White, % | 43.7 | 46.8 | 50.0 | 85.3 | 88.7 | 88.8 | 89.0 | 298 | <0.001 |
| Hispanic, % | 0.0 | 0.2 | 0.3 | 1.3 | 1.7 | 2.0 | 2.3 | 10 | 0.044 |
| Comorbidities, % |  |  |  |  |  |  |  |  |  |
| Depression | 9.3 | 11.0 | 12.7 | 13.7 | 14.3 | 14.3 | 14.3 | 5 | 0.326 |
| Liver disease | 2.3 | 2.7 | 3.0 | 4.3 | 4.3 | 4.3 | 4.3 | 3 | 0.565 |
| Metastatic cancer | 0.3 | 0.3 | 0.3 | 0.3 | 1.3 | 1.5 | 1.7 | 6 | 0.182 |
| Paralysis | 0.0 | 0.2 | 0.3 | 1.0 | 1.3 | 1.5 | 1.7 | 7 | 0.202 |
| Pulmonary disease | 22.0 | 22.2 | 22.3 | 22.3 | 23.3 | 24.0 | 24.7 | 1 | 0.948 |
| Renal disease | 16.0 | 16.8 | 17.7 | 18.0 | 19.0 | 19.0 | 19.0 | 1 | 0.868 |
| Admission source, % |  |  |  |  |  |  |  |  |  |
| Nursing home | 0.3 | 0.5 | 0.7 | 1.0 | 1.3 | 1.7 | 2.0 | 5 | 0.334 |
| Emergency dept. | 2.0 | 4.0 | 6.0 | 8.0 | 13.0 | 16.0 | 19.0 | 60 | <0.001 |
| Broad diagnosis group, % |  |  |  |  |  |  |  |  |  |
| Cardiovascular | 24.7 | 24.7 | 24.7 | 24.7 | 24.7 | 24.7 | 24.7 | 0 | >0.999 |
| Psych / substance | 19.0 | 19.0 | 19.0 | 19.0 | 19.0 | 19.0 | 19.0 | 0 | >0.999 |
| Infection | 16.7 | 16.7 | 16.7 | 16.7 | 16.7 | 16.7 | 16.7 | 0 | >0.999 |
| Gastrointestinal | 8.7 | 8.7 | 8.7 | 8.7 | 8.7 | 8.7 | 8.7 | 0 | >0.999 |
| Respiratory | 12.0 | 12.0 | 12.0 | 12.0 | 12.0 | 12.0 | 12.0 | 0 | >0.999 |
| GU/renal | 4.0 | 4.0 | 4.0 | 4.0 | 4.0 | 4.0 | 4.0 | 0 | >0.999 |
| Other | 15.0 | 15.0 | 15.0 | 15.0 | 15.0 | 15.0 | 15.0 | 0 | >0.999 |
| *Based on the Kruskal-Wallis test for continuous variables and Pearson’s Chi-Squared test for categorical variables | | | | | | | | | |

| **Supplemental Table 5d**: Case-mix variation across matched cohorts for 6 Tier 5 hospitals, using matching run 10 | | | | | | | | | |
| --- | --- | --- | --- | --- | --- | --- | --- | --- | --- |
|  | Range across Facilities | | | | | | | Test Statistic* | *p** |
|  | min | 12.5^th^ percentile | 25^th^ percentile | median | 75^th^ percentile | 87.5^th^ percentile | max |  |  |
| Predicted probability of mortality, median % | 1.1 | 1.1 | 1.1 | 1.1 | 1.1 | 1.2 | 1.3 | 6 | 0.263 |
| Surgical Operations, % | 0.0 | 0.0 | 0.0 | 0.0 | 0.0 | 0.0 | 0.0 |  | >0.999 |
| Demographics |  |  |  |  |  |  |  |  |  |
| Age in years, median | 63 | 63 | 63 | 63 | 63 | 63 | 63 |  | 0.999 |
| Female, % | 2.3 | 3.2 | 3.8 | 4.0 | 4.5 | 6.3 | 9.0 | 18 | 0.006 |
| Black, % | 2.3 | 2.3 | 4.1 | 10.5 | 12.9 | 20.7 | 33.0 | 181 | <0.001 |
| White, % | 61.0 | 72.7 | 79.9 | 82.0 | 84.8 | 87.7 | 91.7 | 102 | <0.001 |
| Hispanic, % | 0.0 | 0.6 | 1.0 | 1.5 | 2.0 | 2.1 | 2.3 | 8 | 0.130 |
| Comorbidities, % |  |  |  |  |  |  |  |  |  |
| Depression | 13.3 | 16.7 | 18.8 | 19.5 | 19.9 | 20.8 | 22.0 | 8 | 0.147 |
| Liver disease | 3.3 | 3.5 | 3.7 | 3.7 | 4.2 | 4.3 | 4.3 | 1 | 0.982 |
| Metastatic cancer | 0.0 | 0.0 | 0.1 | 0.5 | 0.7 | 0.8 | 1.0 | 6 | 0.433 |
| Paralysis | 0.0 | 0.2 | 0.3 | 0.5 | 1.2 | 1.5 | 1.7 | 9 | 0.145 |
| Pulmonary disease | 18.0 | 19.7 | 20.8 | 21.5 | 22.2 | 22.3 | 22.3 | 2 | 0.785 |
| Renal disease | 5.7 | 7.1 | 8.1 | 9.2 | 10.3 | 10.6 | 11.0 | 7 | 0.226 |
| Admission source, % |  |  |  |  |  |  |  |  |  |
| Nursing home | 1.0 | 1.2 | 1.4 | 2.7 | 3.9 | 4.1 | 4.3 | 13 | 0.032 |
| Emergency dept. | 9.7 | 10.1 | 11.1 | 15.0 | 17.7 | 22.6 | 30.3 | 63 | <0.001 |
| Broad diagnosis group, % |  |  |  |  |  |  |  |  |  |
| Cardiovascular | 12.7 | 12.7 | 12.7 | 12.7 | 12.7 | 12.8 | 13.0 | 0 | >0.999 |
| Psych / substance | 42.7 | 42.9 | 43.0 | 43.0 | 43.0 | 43.0 | 43.0 | 0 | >0.999 |
| Infection | 12.7 | 12.7 | 12.7 | 12.7 | 12.7 | 12.7 | 12.7 | 0 | >0.999 |
| Gastrointestinal | 6.7 | 6.7 | 6.7 | 6.7 | 6.7 | 6.7 | 6.7 | 0 | >0.999 |
| Respiratory | 8.7 | 8.7 | 8.7 | 8.7 | 8.7 | 8.7 | 8.7 | 0 | >0.999 |
| GU/renal | 3.7 | 3.7 | 3.7 | 3.7 | 3.7 | 3.7 | 3.7 | 0 | >0.999 |
| Other | 12.7 | 12.7 | 12.7 | 12.7 | 12.7 | 12.7 | 12.7 | 0 | >0.999 |
| *Based on the Kruskal-Wallis test for continuous variables and Pearson’s Chi-Squared test for categorical variables | | | | | | | | | |

**Supplemental Figure1:** Hospital rankings by template matching run 10 versus run 8


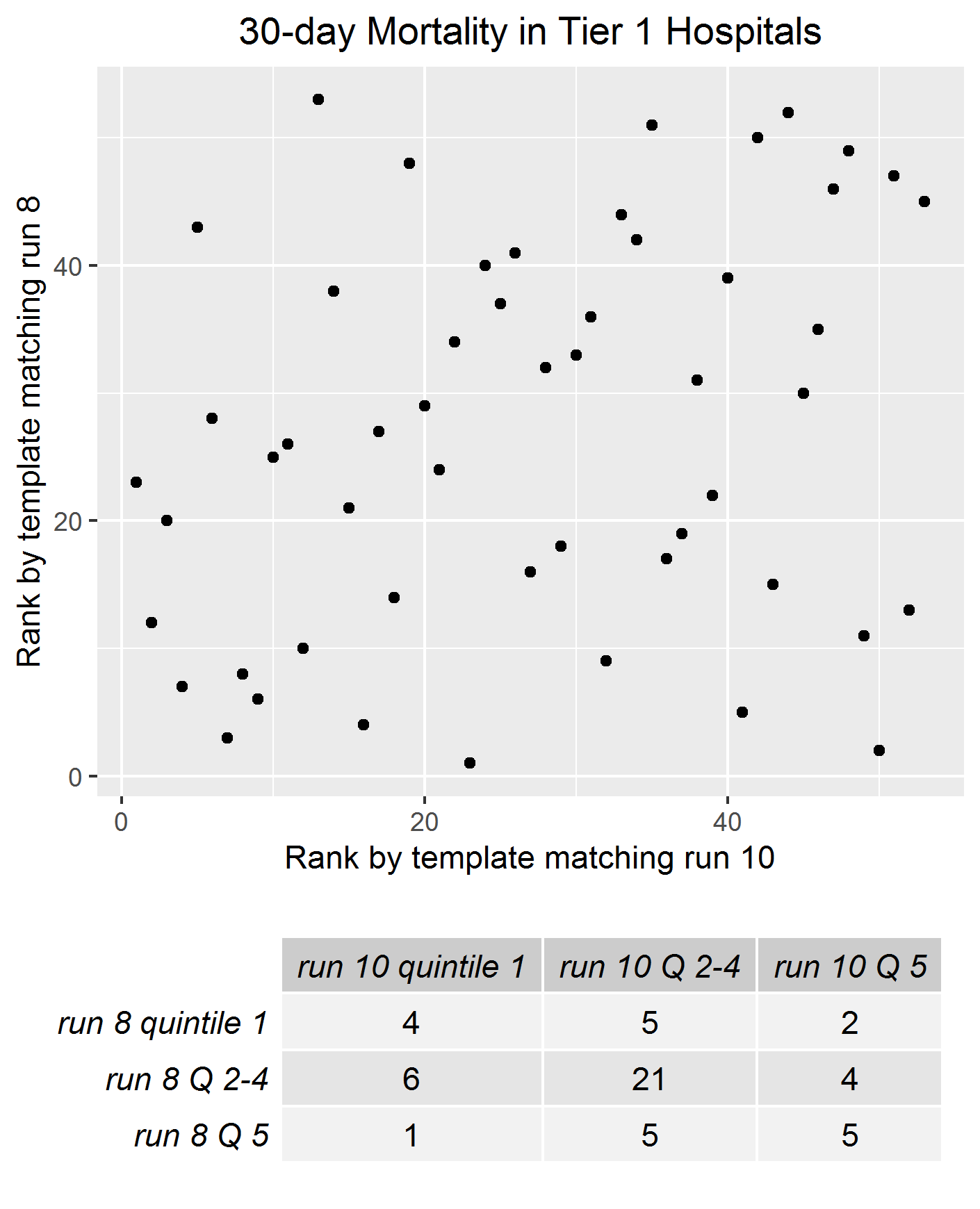

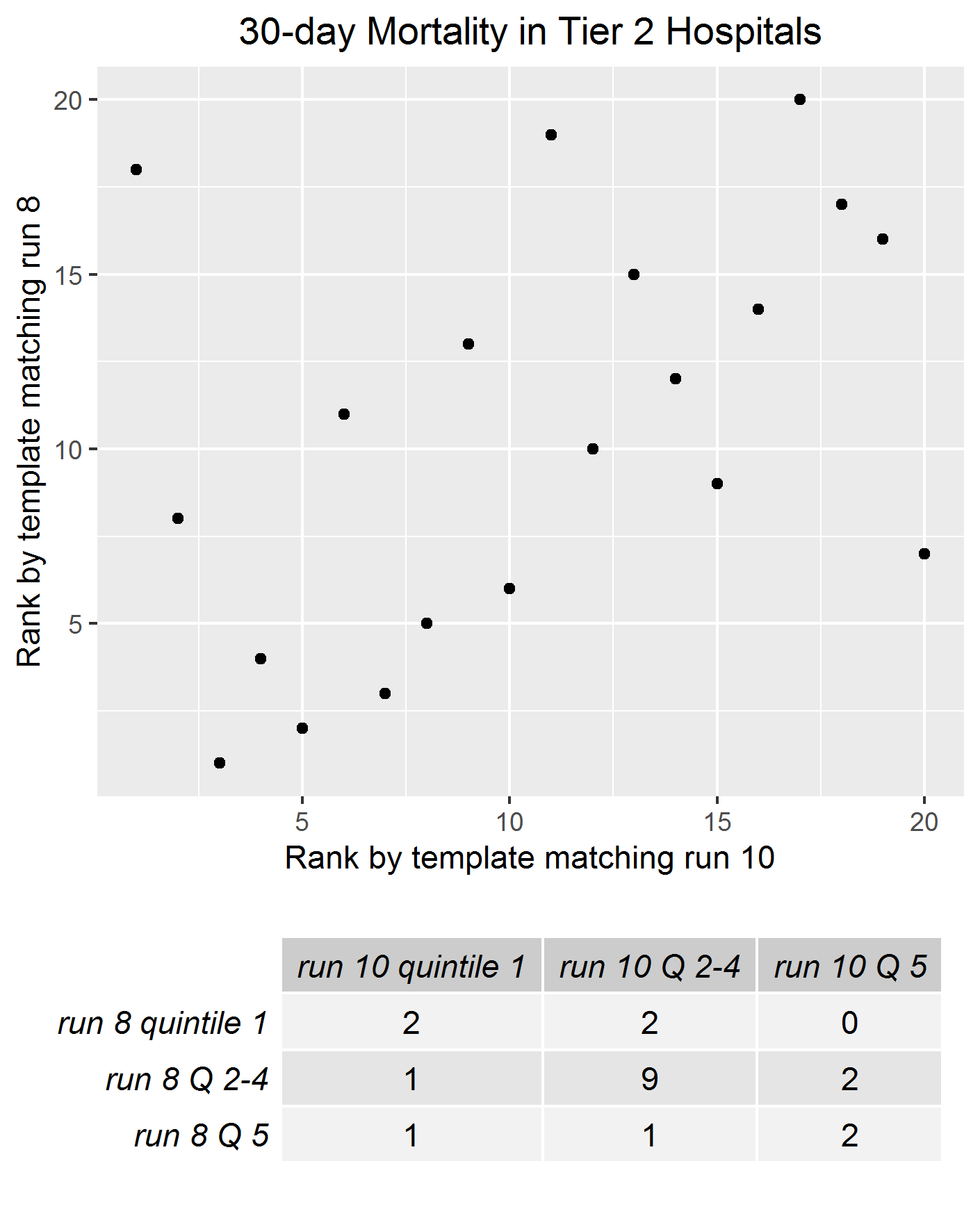

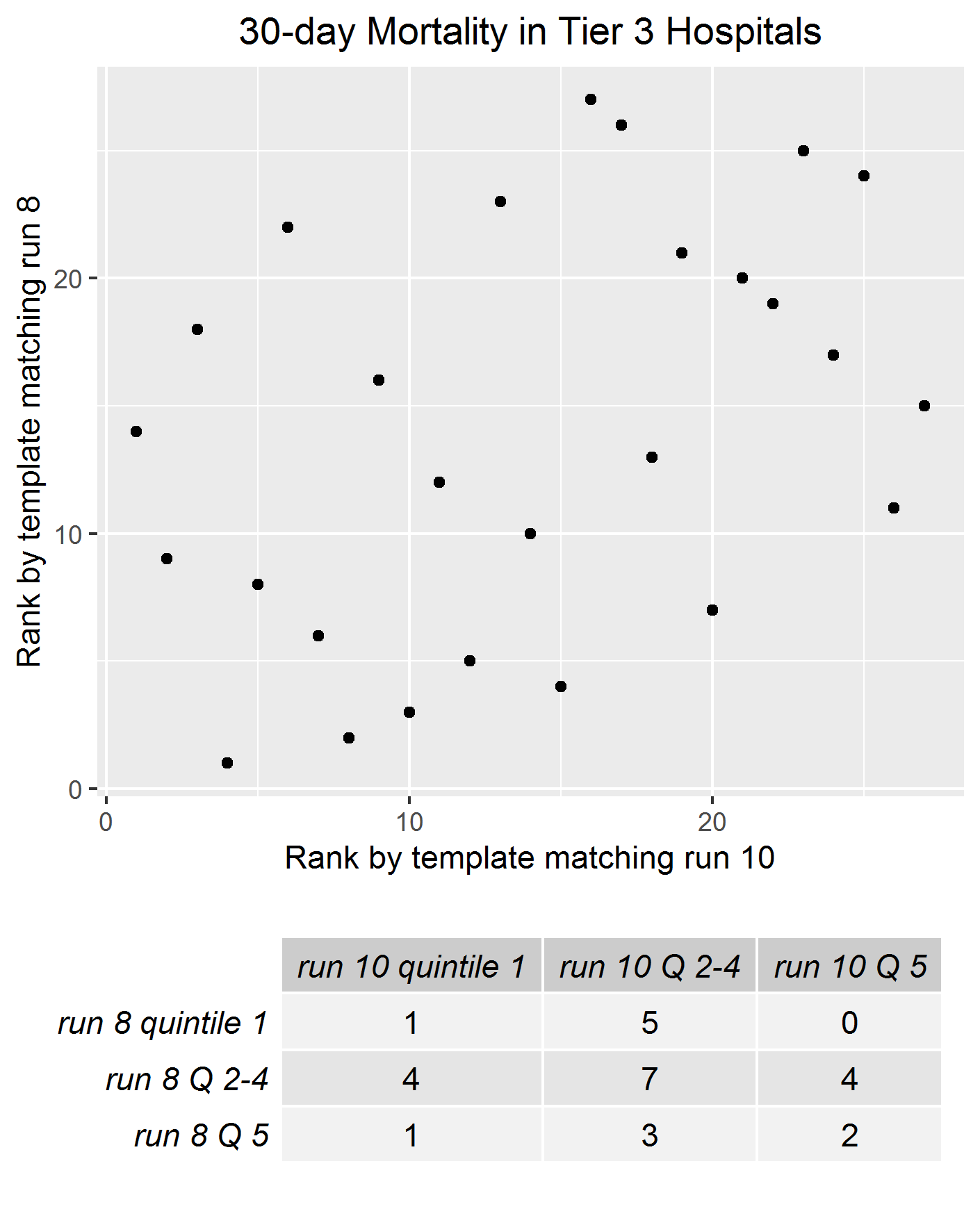


**Legend**: Plot of 55 tier 1 hospital rankings (Panel A), 20 tier 2 hospital rankings (Panel B), and 31 tier 3 hospital rankings (Panel C) when using template matching run 10 (x-axis) versus template matching run 8 (y-axis).

**Supplemental Figure 2:** Hospital rankings by template matching (run 10) versus unmatched logistic regression

**Panel A, B, C**


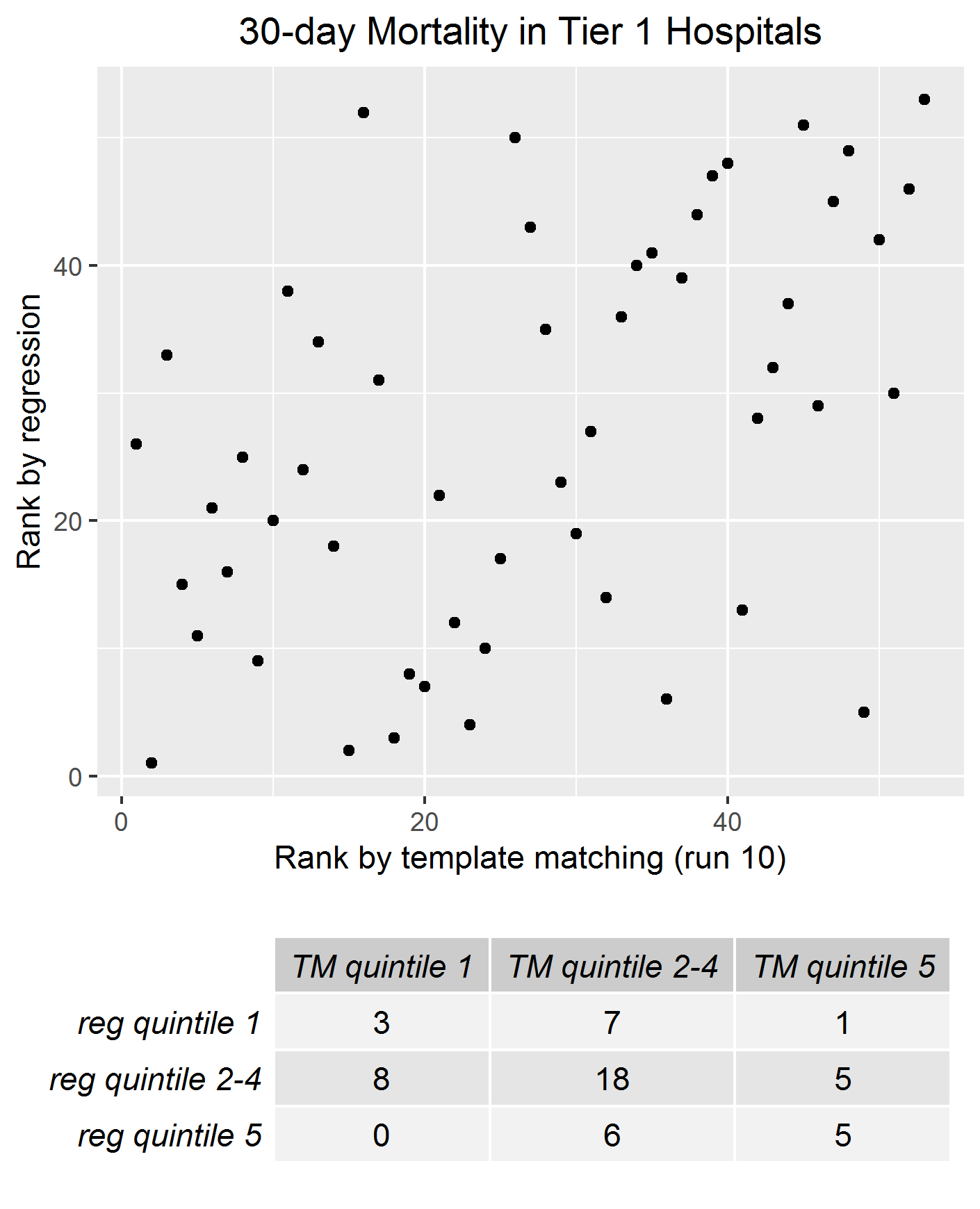

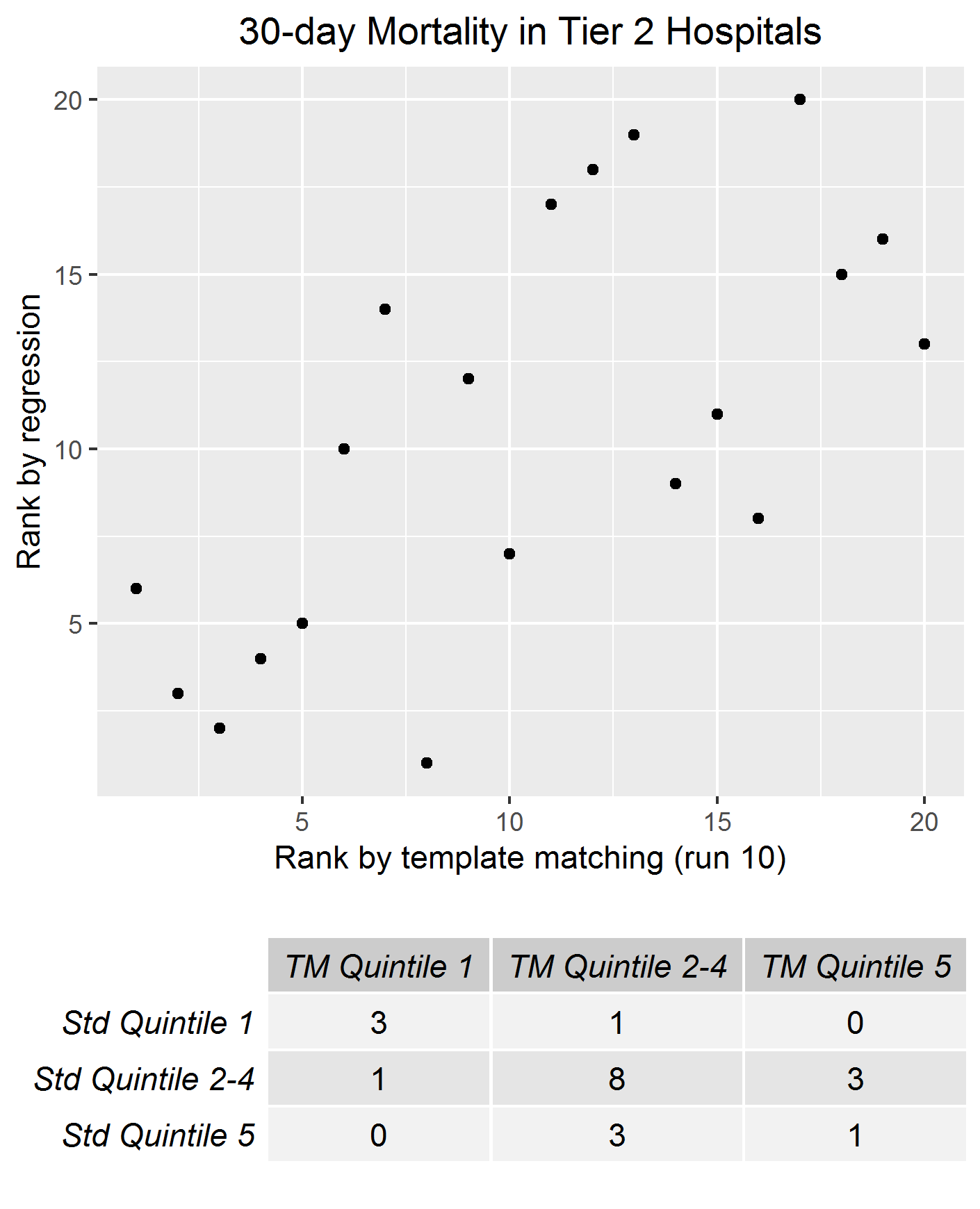

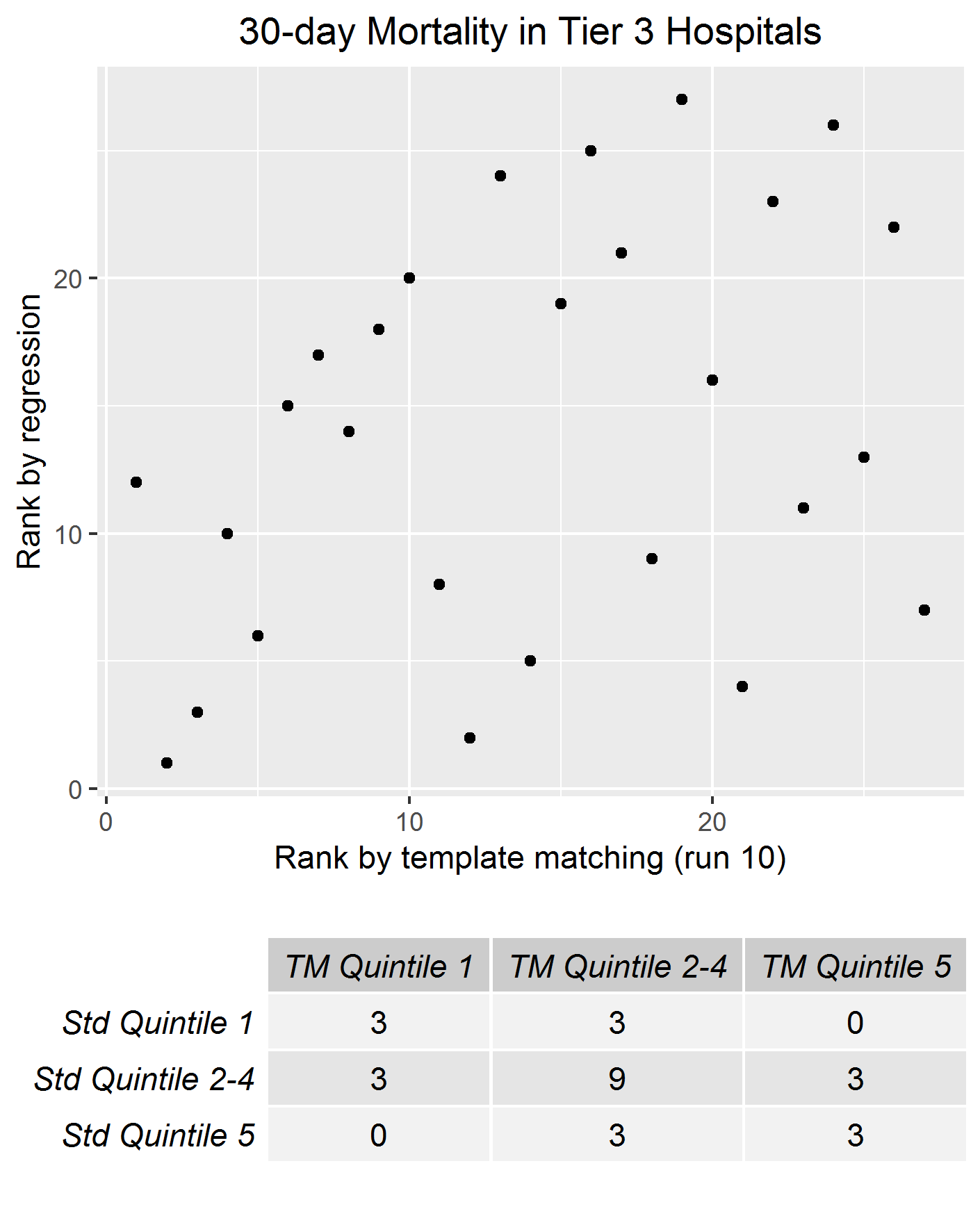


**Legend**: Plot of 55 tier 1 hospital rankings (Panel A), 20 tier 2 hospital rankings (Panel B), and 31 tier 3 hospital rankings (Panel C) when using template matching (x-axis) versus hierarchical risk-adjusted regression (y-axis).

**Supplemental Figure 3:** Hospital rankings by template matching (run 8) versus regression

**Panel A, B, C**


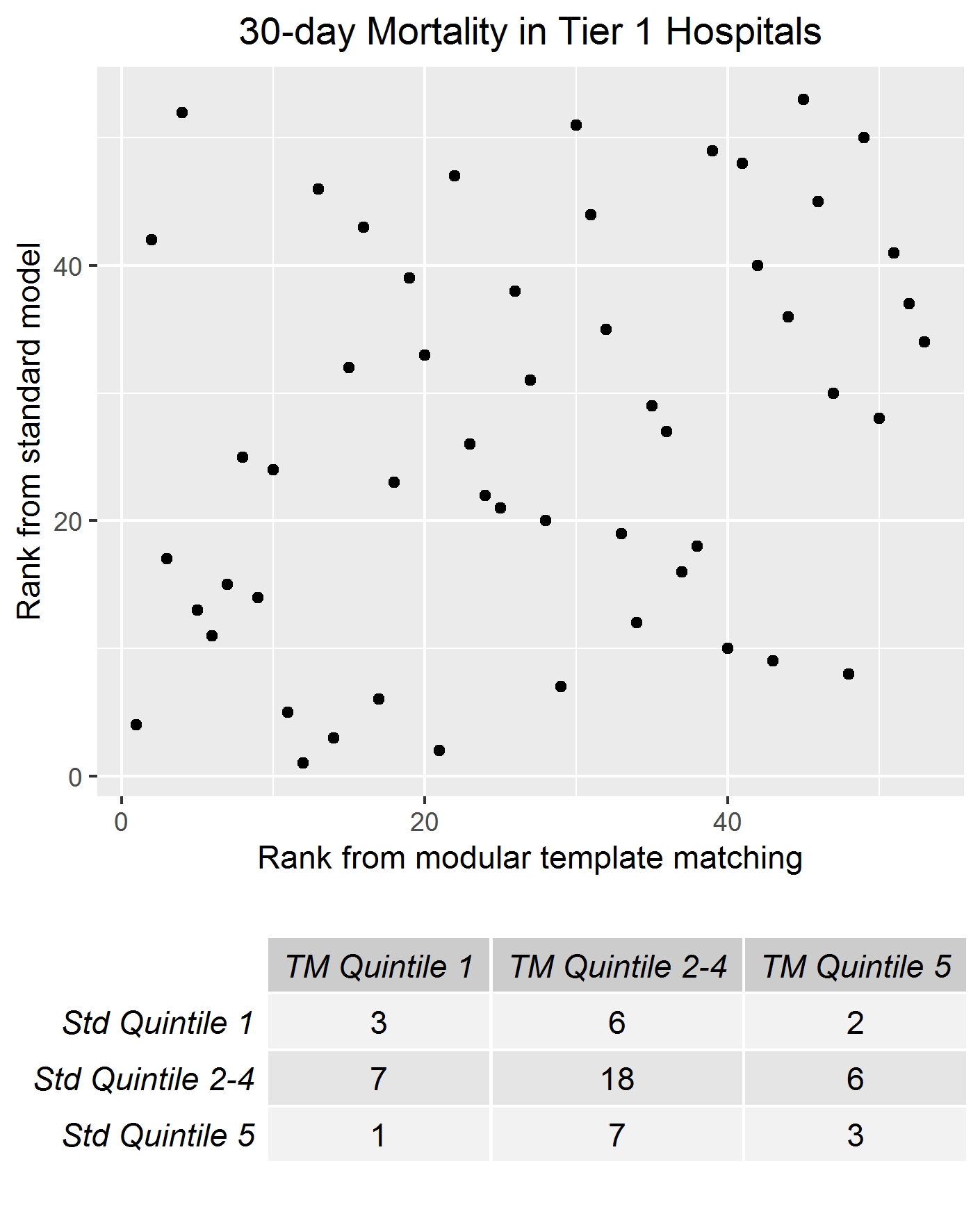

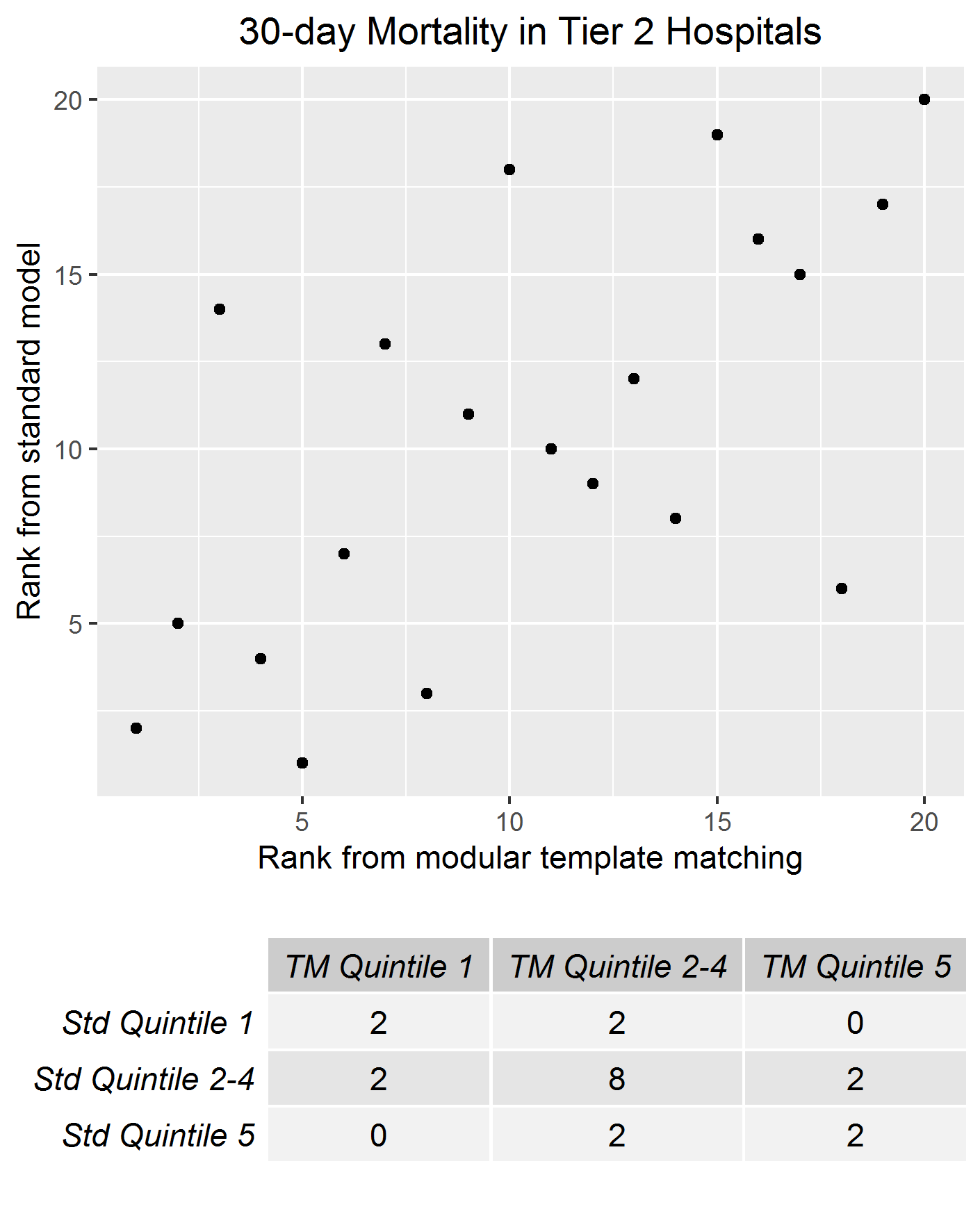

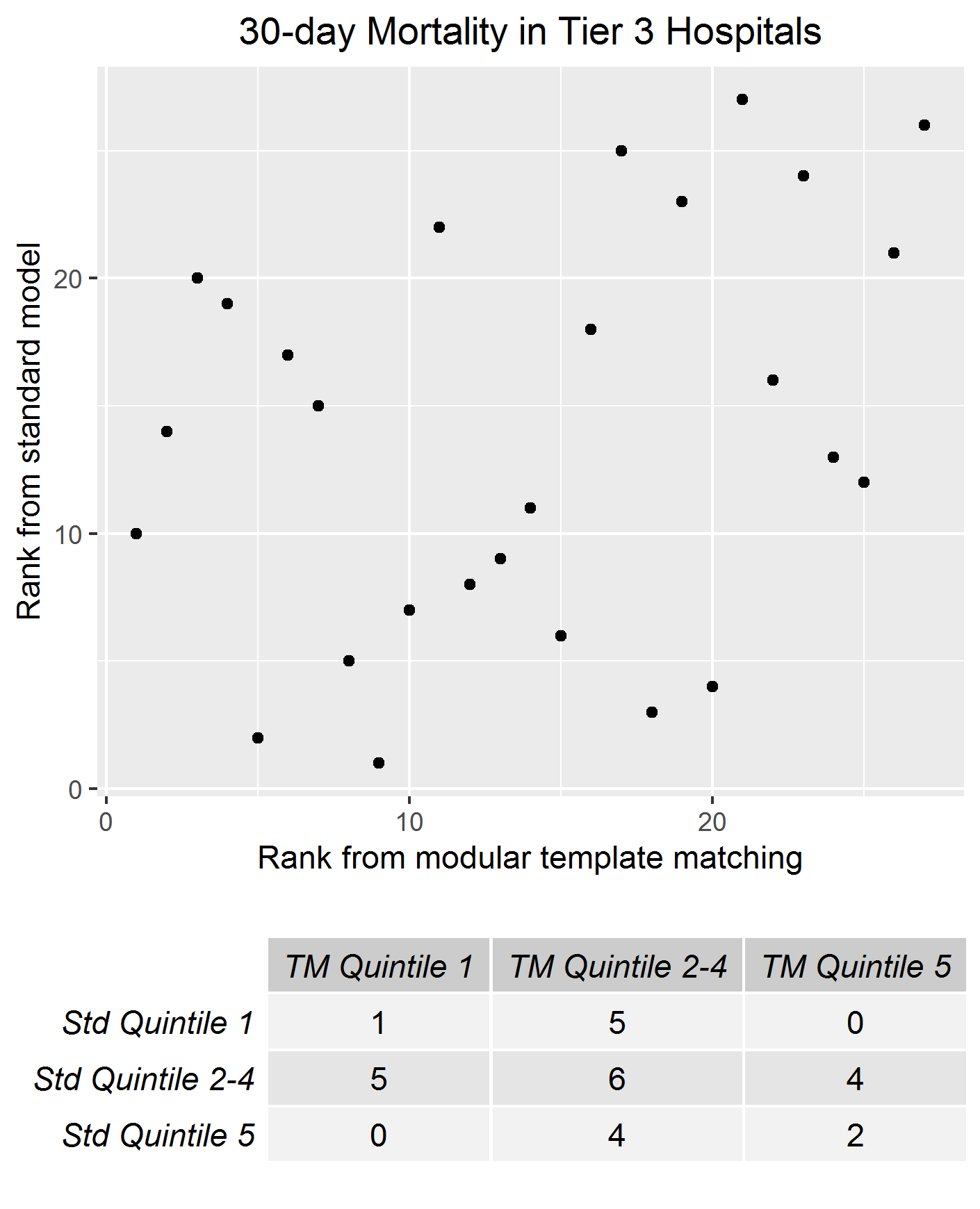


**Legend**: Plot of 55 tier 1 hospital rankings (Panel A), 20 tier 2 hospital rankings (Panel B), and 31 tier 3 hospital rankings (Panel C) when using template matching (x-axis) versus hierarchical risk-adjusted regression (y-axis).

**Appendix 1: Description of VA computing environment**

The VA Informatics and Computing Infrastructure (VINCI) is a Department of Veterans Affairs (VA) Health Services Research & Development (HSR&D) resource center that provides a secure, central analytic platform for performing research and supporting clinical operations activities. It is a partnership between the VA Office of Information Technology (OI&T) and the Veterans Health Administration Office of Research and Development (VHA ORD). VINCI includes a cluster of servers for securely hosting suites of databases integrated from select national VA data sources. VINCI servers for data, applications and virtual sessions are physically located at the VA Austin Information Technology Center (AITC), located in Austin, Texas. This secure enclave with 105 high-performance servers and 1.5 petabytes of high-speed data storage has multiple layers of security and disaster recovery to prevent data loss.
